# Identification of Newly Committed Pancreatic Cells in the Adult Mouse Pancreas

**DOI:** 10.1038/s41598-017-17884-z

**Published:** 2017-12-13

**Authors:** Mairobys Socorro, Angela Criscimanna, Patricia Riva, Manuj Tandon, Krishna Prasadan, Ping Guo, Abhinav Humar, Sohail Z. Husain, Steven D. Leach, George K. Gittes, Farzad Esni

**Affiliations:** 1 Department of Surgery, University of Pittsburgh Medical Center, One Children’s Drive, 4401 Penn Avenue, Rangos Research Center, Pittsburgh, PA 15244 USA; 2 Division of Pediatric General and Thoracic Surgery, Children’s Hospital of Pittsburgh, University of Pittsburgh Medical Center, One Children’s Drive, 4401 Penn Avenue, Rangos Research Center, Pittsburgh, PA 15244 USA; 3 Department of Pediatrics, Children’s Hospital of Pittsburgh, University of Pittsburgh Medical Center, Pittsburgh, PA 15244 USA; 4 Rubenstein Center for Pancreatic Cancer Research, Memorial Sloan Kettering Cancer Center, New York, NY 10065 USA; 5 Department of Developmental Biology, University of Pittsburgh, Pittsburgh, PA 15244 USA; 6 Department of Microbiology and Molecular Genetics, University of Pittsburgh, Pittsburgh, PA 15244 USA; 7 University of Pittsburgh Cancer Institute, Pittsburgh, PA 15123 USA; 8 Present Address: Department of Orthopaedic Surgery, University of Texas Health Science Center at Houston, 1881 East Road, 3SCR6.4621, Houston, Texas 77054 USA

## Abstract

Multipotent epithelial cells with high Aldehyde dehydrogenase activity have been previously reported to exist in the adult pancreas. However, whether they represent true progenitor cells remains controversial. In this study, we isolated and characterized cells with ALDH activity in the adult mouse or human pancreas during physiological conditions or injury. We found that cells with ALDH activity are abundant in the mouse pancreas during early postnatal growth, pregnancy, and in mouse models of pancreatitis and type 1 diabetes (T1D). Importantly, a similar population of cells is found abundantly in healthy children, or in patients with pancreatitis or T1D. We further demonstrate that cells with ALDH activity can commit to either endocrine or acinar lineages, and can be divided into four sub-populations based on CD90 and Ecadherin expression. Finally, our *in vitro* and *in vivo* studies show that the progeny of ALDH1^+^/CD90^−^/Ecad^−^ cells residing in the adult mouse pancreas have the ability to initiate Pancreatic and duodenal homeobox (Pdx1) expression for the first time. In summary, we provide evidence for the existence of a sortable population of multipotent non-epithelial cells in the adult pancreas that can commit to the pancreatic lineage following proliferation and mesenchymal to epithelial transition (MET).

## Introduction

Despite advancements in islet transplantation and immune therapies, an effective cell replacement therapy for curing T1D is still needed. One attractive approach would be to generate new β-cells from progenitor-like cells residing within the adult pancreas. However, the existence of such cells remains controversial. The pancreas is an endoderm-derived organ, and thus the search for multipotent cells present in the adult pancreas has been greatly influenced by the assumption that potential progenitor-like or facultative stem cells should originate from the pool of epithelial PDX1-positive cells in the developing pancreas. Given the importance of mesenchymal-to-epithelial transition during embryonic implantation, organogenesis and somitogenesis^1–3^, we sought to challenge the epithelial origin dogma and instead looked for potential sources for multipotent cells outside the pancreatic parenchyma.

In the adult mouse pancreas, β-cell regeneration under physiological conditions occurs through β-cell self-duplication^4,5^, and accordingly considerable effort has been put into finding ways to stimulate endogenous β-cell proliferation^6^. On the other hand, in the regenerating pancreas, depending on the injury model, it appears that new β-cells can arise from cells either residing within the ducts^7–11^, or in proximity to the ductal network^10^. However, the notion of progenitor-like cells existing within pancreatic ducts have been challenged by studies showing that lineage-labeled ductal cells do not contribute to new acinar or endocrine cells during normal growth or after injury^12,13^. Nevertheless, recent reports using pancreatic ducts to generate acinar or endocrine cells have focused the search for progenitor cells to within or in proximity to ductal structures^14–16^.

ALDH is a generic designation for a closely related superfamily of 19 human genes^17,18^. Stem or progenitor cells from different lineages such as hematopoietic, mammary, prostate, mesenchymal, neuronal, and skeletal muscle display high ALDH activity^18–28^. The enzymatic activity of ALDH allows isolation by fluorescence-activated cell sorting (FACS) using a fluorogenic ALDH substrate known as Aldefluor. Thus, Aldefluor has been used to enrich populations of stem and progenitor cells from a variety of adult tissues^17,18^. Leach and colleagues used Aldefluor to sort central acinar/terminal duct cells from the adult mouse pancreas, and showed that the isolated Aldefluor^+^ cells were able to differentiate into both endocrine and acinar cells when cultured as “pancreatospheres”, or when co-cultured with embryonic pancreatic explants *in vitro* ^29^.

Here, we have identified a novel population of multipotent Aldefluor^+^/CD90^−^/Ecad^−^ mesenchymal cells residing within the adult pancreas that appears to not be derived from the PDX1^+^ lineage. These cells proliferate during post-natal growth, pregnancy, and in pathological conditions such as pancreatitis and diabetes. These Aldefluor^+^/CD90^−^/Ecad^−^ cells undergo a subsequent mesenchymal to epithelial transition prior to committing to a pancreatic lineage by expressing Pdx1 for the first time.

## Results

### Expansion of Aldefluor^+^ cells in mouse and human pancreas

By using the Aldefluor-assay, Leach and colleagues were able to identify a population of cells in the adult mouse pancreas that expressed embryonic pancreatic markers, formed spheres and could differentiate into endocrine and acinar cells^29^. To study ALDH-expressing cells under different physiological or pathological conditions we performed enzymatic digestion of the mouse pancreas, followed by multiple rounds of filtering based on cell size^30^. This protocol provided us with a starting pool of approximately 7 × 10^6^ cells per pancreas highly enriched for viable Dolichos biflorus agglutinin-posiitve (DBA) duct cells (Ecadherin^+^/DBA^+^) (Supplementary Fig. 1A). Using this isolation method, and consistent with a previous report^29^, we determined the percentage of Aldefluor^+^ cells in the adult wild type pancreas to be between 0.5–1% of the duct-enriched cells (Supplementary Fig. 1B). Quantitative RTPCR analyses confirmed the lack of acinar or β-cell contamination, as no insulin or amylase transcript could be detected in the sorted Aldefluor^+^ cell pool (Supplementary Fig. 1C).

**Figure 1:**
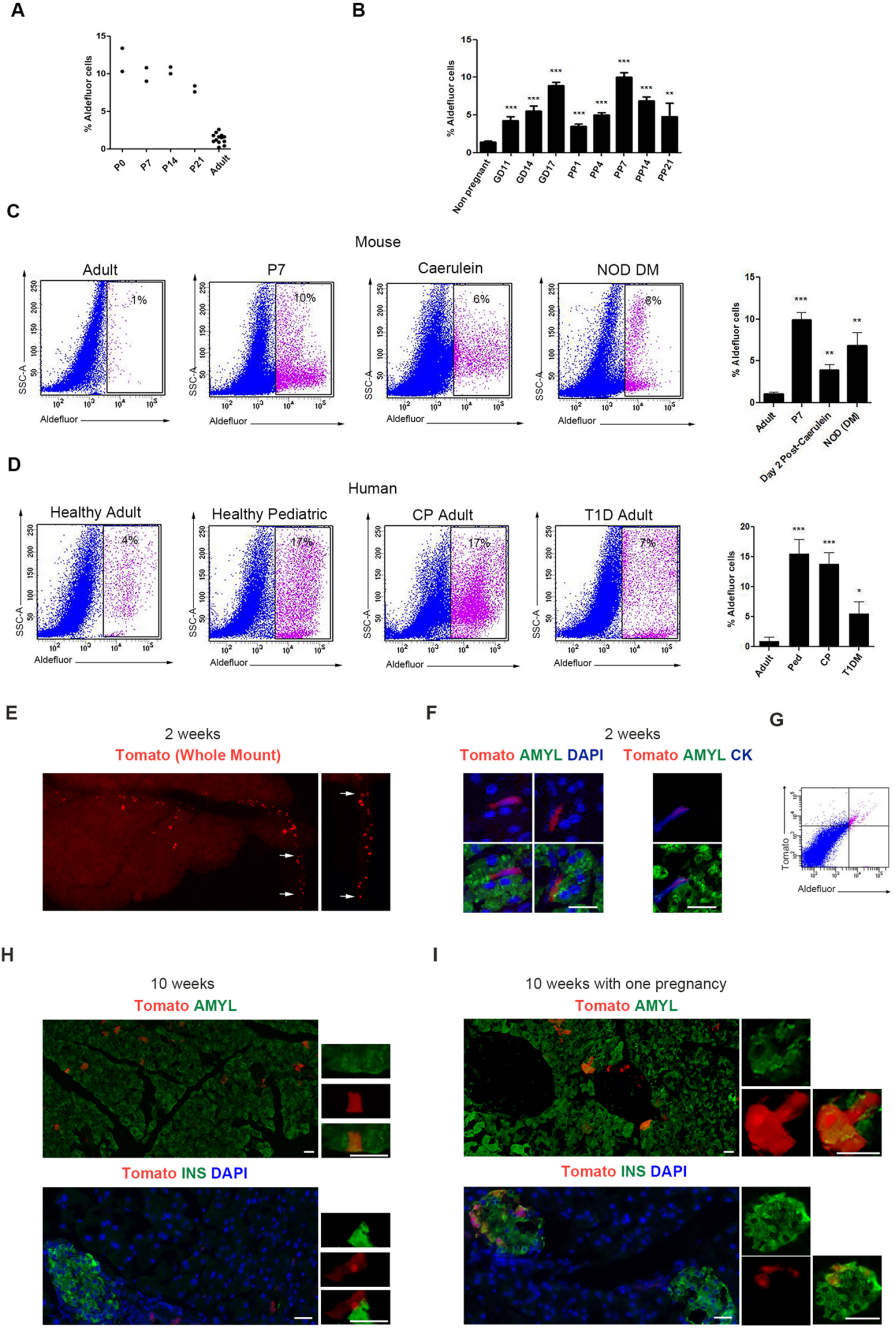
Cells with ALDH-activity are abundant in the pancreas, and can differentiate into acinar and endocrine cells. (**A**,**B**) Quantification of cells with ALDH-activity (Aldefluor) in the pancreas of (A) post-natal mice (n = 2, pooled samples from 7–10 pups for each time point), or adult non-pregnant (n = 13) (**B**) pregnant (GD11 n = 8, GD14 n = 14, GD17 n = 44) or postpartum dams (PP1 n = 31, PP4 n = 37, PP7 n = 12, PP14 n = 5, PP21 n = 4). Aldefluor^+^ cells are highly abundant before weaning in neonatal mice, as well as in pregnant and postpartum dams. (**C**) Representative flow cytometric analysis and quantification of Aldefluor^+^ cells in the pancreas of adult (n = 13), P7 (n = 2, pooled samples from 7–10 pups), caerulein-treated (n = 5) or diabetic NOD (n = 4) mice, or (**D**) healthy adult (n = 5), healthy pediatric (n = 3), chronic pancreatitis (n = 9), or adult T1D (n = 3) humans. Aldefluor^+^ cells isolated from human pancreas display a similar expansion pattern to their mouse counterparts. (**E**) Whole-mount detection of tomato-red in the pancreas of R26^Tomato^ mice two weeks after Aldh1a1-Cre viral infusion. Labeled cells can be found within or outside the parenchyma (arrows). (**F**) Immunofluorescent staining for amylase and cytokeratin shows that tomato-red labeled cells within the parenchyma are located at the terminal duct-acini junction and are express amylase^−^/cytokeratin^+^. (**G**) Flow cytometric analysis of tomato-red expression in Aldefluor^+^ cells from the pancreas of Aldh1a1-Cre viral infused R26^Tomato^ mice. (**H**,**I**) Detection of tomato-red, with amylase, or insulin in the pancreas of R26^Tomato^ mice ten weeks after Aldh1a1-Cre viral infusion without (**H**) or with one pregnancy (**I**). Insets (**H**) show Tomato^+^/amylase^+^, or Tomato^-^/insulin^+^ cells, and insets in (**I**) show Tomato^+^/amylase^+^, or Tomato^+^/insulin^+^ cells. Results in (**A**) show the individual data points for each sample, and in (**B**) are expressed as mean ± standard error of the mean (SEM) and analyzed statistically by Student’s t-test, **p < 0.01, ***p < 0.001. P0-P21: post-natal days 0 to 21; GD11-GD17: gestational days 11 to 17; PP1-PP21: postpartum days 1 to 21; DM: diabetes mellitus; CP: chronic pancreatitis; T1D: type 1 diabetes. Bars 20 μm.

ALDH-expressing cells are present in both embryonic and the adult mouse pancreas, and are markedly expanded during caerulein-induced chronic pancreatitis (CP)^29^. The expansion of ALDH^+^ cells during CP led us to study the presence of these cells in other physiological or pathological conditions involving increased pancreatic work-load. Post-natal pancreas is associated with extensive growth of the gland. Therefore, we next evaluated the percentage of Aldefluor^+^ cells in the pancreas at serial time points between birth and 8 weeks of age (Fig. 1A, Supplementary Fig. 1D). Of note, the percentage of Aldefluor^+^ cells appeared to be greater early during post-natal growth of the pancreas, and then started to gradually decline around the time of weaning. Since pregnancy in mice entails a non-pathologic increase in β-cell numbers, we next studied these cells in the dam pancreas during pregnancy or postpartum until weaning, and found that the total number of Aldefluor^+^ cells under these conditions was significantly higher compared to non-pregnant age-matched mice (Fig. 1B, Supplementary Fig. 1E).

Interestingly, in addition to pregnancy, lactation, and early post-natal growth, the Aldefluor^+^ cell population also expanded following caerulein-induced acute pancreatitis, as well as in non-obese diabetic (NOD) mice after the onset of overt diabetes (Fig. 1C). Encouraged by our findings in the mouse pancreas, we sought to study cells with ALDH-activity in the human pancreas. To do so, we obtained cells from the pancreas of healthy pediatric, healthy adult or adult T1D donors, as well as patients with CP undergoing total pancreatectomy for autologous islet transplantation. Aldefluor^+^ cells isolated from human pancreata showed remarkably similar patterns of expansion to their mouse counterparts (Fig. 1D).

### Aldh1a1^+^ cells can give rise to acinar and β-cells *in vivo*

ALDH-positive cells can differentiate into both acinar and endocrine cells *in vitro* ^29^. To determine to what extent the Aldefluor^+^ cells can give rise to other pancreatic cell types *in vivo*, we first created an AAV6 viral vector to express Cre-recombinase under the Aldh1a1-promoter^31^ (AAV6-AldhCre). In an effort to obtain direct, permanent labeling of pancreatic cells without the need for a tamoxifen-inducible cre-system, a viral pancreatic duct infusion system has been recently developed^32^. For viral lineage tagging, we infused AAV6-AldhCre virus into the pancreatic ducts of 8 week old R26^Tomato^ mice. To evaluate the specificity of the promoter, the mice were sacrificed two weeks after the viral infusion (Fig. 1E). Labeled cells were amylase-negative, but expressed cytokeratin and were localized at the acinar-ductal junction, suggesting that these may be the centro-acinar-cells (Fig. 1F). Surprisingly, we could also find some labeled cells that were scattered outside the pancreatic parenchyma and in between the lobes (Fig. 1E). The exclusive localization of Aldefluor^+^ cells with Tomato^+^ cells further confirmed our ability to target cells with ALDH-activity (Fig. 1G). Next, to look at cell lineages arising from these ALDH1a1^+^ cells, pancreata were harvested ten weeks after viral infusion, and could detect acinar cells (but not endocrine cells) expressing Red-Tomato (Fig. 1H). Interestingly, in a parallel experiment, when mice were harvested ten weeks after infusion, but with one round of pregnancy and weaning during that window of time, in addition to acinar cells, strikingly now some β-cells were also labeled (Fig. 1I). These data show that cells with ALDH-activity contribute normally to maintain acinar mass, but more importantly, following expansion during pregnancy, can give rise to β-cells. To determine when the transformation into β-cells occurred, we next analyzed the AAV6-AldhCre injected R26^Tomato^ reporter dams at gestational day 15 as well as postpartum days 1, 7, and 14. Here, and in accordance with a previous report showing that β-cell neogenesis does not occur during pregnancy^33^, we could detect labeled β-cells in the pancreas of virus-injected reporter dams first on postpartum day 14 (data not shown). Together, our results show that Aldh1a1^+^ cells expand during pregnancy, and can transform into β-cells before weaning.

### Identification of four sub-groups within the total Aldefluor^+^ cell population in the adult pancreas

**Figure 2:**
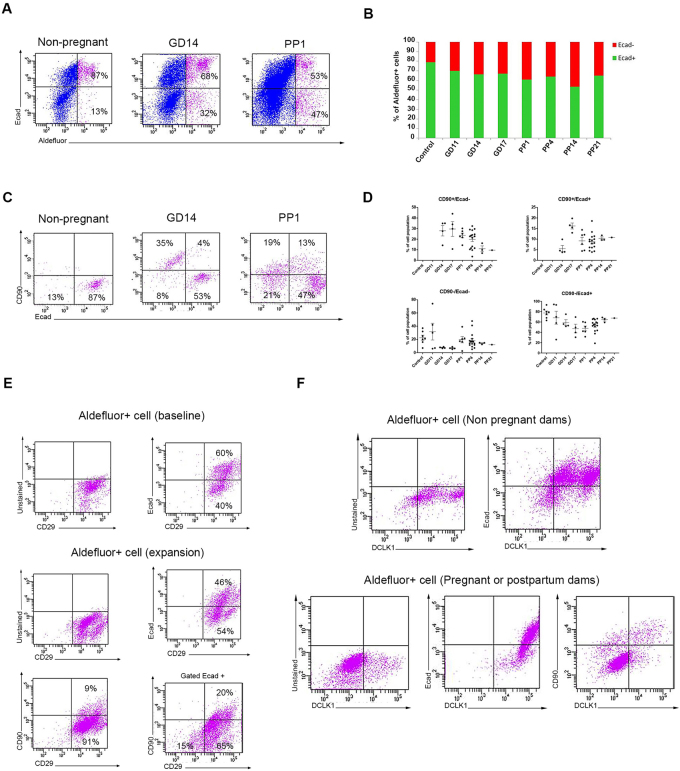
Aldefluor^+^ cells can be separated into four sub-populations. (**A**) Representative flow cytometric analysis of Ecadherin expression in Aldefluor^+^ cells from the pancreas of non-pregnant (n = 7), gestational day 14 pregnant (n = 14), or postpartum day 1 dams (n = 25). (**B**) Median percentages of Ecad^+^ (green) and Ecad^−^ (red) cells within the Aldefluor^+^ population in the pancreas of non-pregnant (n = 7), pregnant (GD11 n = 5, GD14 n = 14, GD17 n = 24) or postpartum dams (PP1 n = 25, PP4 n = 26, PP7 n = 12, PP14 n = 5, PP21 n = 4). (**C**) Representative flow cytometric analysis of Ecadherin and CD90 expression in Aldefluor^+^ cells from the pancreas of non-pregnant (n = 7), gestational day 14 pregnant (n = 4), or postpartum day 1 dams (n = 6). (**D**) Quantification of the percentage of the four sub-populations with Aldefluor^+^ cells isolated from the pancreas of non-pregnant, pregnant, or postpartum dams at indicated stages. Results are expressed as mean ± standard error of the mean (SEM). non-pregnant (n = 7), pregnant (GD11 n = 5, GD14 n = 4, GD17 n = 4), postpartum dams (PP1 n = 6, PP4 n = 16, PP14 n = 3, PP21 n = 1). The corresponding data for (**B**) is presented in Supplementary Fig. 2, and for (**A**,**C** and **D**) is presented in Supplementary Fig. 4. (**E**) Representative flow cytometric analysis of CD29, Ecadherin and CD29, or CD90 and CD29 expression in Aldefluor^+^ cells from the pancreas of non-pregnant (baseline), or during pregnancy or postpartum Aldefluor^+^ cell expansion. Representative flow cytometric analysis of CD29 and CD90 expression in Aldefluor^+^/Ecad^+^ cells (lower right panel). (**F**) Representative flow cytometric analysis of DCLK1, or DCLK1 and Ecadherin expression in Aldefluor^+^ cells from the pancreas of non-pregnant (baseline), or postpartum (expansion) dams showing that DCLK1 is expressed specifically by the CD29^+^/CD90^−^/Ecad^+^ cells (n = 5).

It has been reported that the Aldefluor^+^ population in the pancreas of non-pregnant wild type mice are mainly epithelial (Ecadherin^+^) and are located at the acinar-ductal junctions^29^. However, our findings in pregnant and lactating dams show a dramatic amplification of a previously undescribed Aldefluor^+^/Ecadherin^−^ population, which was equally prevalent in the head, body and tail portions (Fig. 2A, Supplementary Fig. 2A,B). More detailed analyses of the ratio between the Ecad^+^ and Ecad^−^ cells within the Aldefluor^+^ population during pregnancy and lactation confirmed that there was a relative greater expansion of Ecadherin^−^ cells compared to expansion of Ecadherin^+^ cells (Fig. 2B, Supplementary Fig. 2C). Notable, the expansion of this Aldefluor^+^/Ecad^−^ population was not due to an influx of F4–80^+^/CD11b^+^ macrophages or contamination by CD31^+^ endothelial cells, as those cells were not detected within the Aldefluor^+^ population (Supplementary Fig. 2D). A similar expansion of Aldefluor^+^ cells in general, and Aldefluor^+^/Ecadherin^−^ in particular could be detected in the pancreas of pre-weaning pups, caerulein-treated mice, and diabetic NOD mice, as well as in healthy children, type 1 diabetics, and patients with pancreatitis (Supplementary Fig. 2E,F).

To better characterize these two Aldefluor^+^ populations, we looked for expression of markers reported to be present in multipotent mesenchymal or epithelial cells. However, the Aldefluor^+^ cells isolated from non-pregnant or pregnant dams did not express the stem cell markers Sca-1, CD44, CD45, CD105 or CD133 (Supplementary Fig. 3). CD90 is a marker of some cancer cells and normal stem cells, typically those that are mesenchyme-derived, in various tissues^34^. FACS-based characterization of Aldefluor^+^ cells showed that while none or very few CD90^+^ cells could be detected in the non-pregnant pancreas, a CD90^+^ subpopulation emerged during pregnancy, which was sustained throughout lactation (Fig. 2C, Supplementary Fig. 4A). Using CD90 and Ecadherin as markers, we were able to separate the Aldefluor^+^ cells into four important subgroups: ALDH1^+^/CD90^−^/Ecad^−^ (hereby defined as CD90^−^/Ecad^−^ cells), ALDH1^+^/CD90^+^/Ecad^−^ (CD90^+^/Ecad^−^ cells), ALDH1^+^/CD90^+^/Ecad^+^ (CD90^+^/Ecad^+^ cells), and ALDH1^+^/CD90^−^/Ecad^+^ (CD90^−^/Ecad^+^ cells). The total number and percentage of each sub-population changed at different time points during pregnancy and lactation (Fig. 2D, Supplementary Fig. 4B,C). The percentage of mice having Aldefluor^+^/CD90^+^ cells in the pancreas increased concomitantly with gestational stage, and peaked toward the end of gestation, when Aldefluor^+^/CD90^+^ cells were present in 75–80% of dams (Supplementary Fig. 4D). The number of mice having pancreatic Aldefluor^+^/CD90^+^ cells declined during the first postpartum days to only 16 of 27 mice (59%) (Supplementary Fig. 4D).

The Aldefluor^+^ cells do not represent a homogenous population, with there being at least four sub-populations based on Ecadherin and CD90 expression. To further evaluate the level of heterogeneity within the four Aldefluor^+^ subpopulation described in Fig. 2, we stained the cells with CD90 and Ecadherin and subsequently studied their Aldefluor intensity, size and granularity (Supplementary Figure 5). The CD90^−^/Ecad^−^ and the CD90^+^/Ecad^−^ cells contained mainly smaller Aldefluor^+^ cells with low granularity, whereas CD90^+^/Ecad^+^ cells were larger but displayed similar low granularity. On the other hand, the CD90^+^/Ecad^+^ fraction consisted of a more heterogeneous population of cells. These data indicate that the heterogeneity among the Aldefluor^+^ cells is not only reflected by the presence of specific surface antigens, but also by their size and granularity.

CD90 is typically considered to be a mesenchymal marker, so its co-expression with the epithelial marker Ecadherin in CD90^+^/Ecad^+^ cells was intriguing, as it suggested that cells in this Aldefluor^+^ sub-population may exist in a “semi-epithelial” state. Moreover, all Aldefluor^+^ cells, regardless of Ecadherin expression at baseline expressed CD29 (Fig. 2E, top panel). Similarly, CD29 could be found in all Aldefluor^+^ cells during pregnancy or lactation (Fig. 2E, middle panel). Additionally, when Aldefluor^+^ cells were gated for Ecadherin expression, CD29^+^ cells could be detected within both CD90^+^ as well as CD90^−^ cell populations (Fig. 2E, lower panel), indicating that CD29 is expressed by all four Aldefluor^+^ populations. A recent report demonstrates that Doublecortin like kinase 1 (DCLK1) is expressed in a subset of acinar or duct cells that display progenitor cell features^35^. Notably, DCLK1 can be detected in Aldefluor^+^/Ecad^+^ cells at baseline, and in CD90^−^/Ecad^+^ cells during pregnancy and lactation (Fig. 2F). Thus, while the recently reported DCLK1^+^ quiescent progenitor cells are distinct from Aldefluor^+^ cells found in CD90^−^/Ecad^−^, CD90^−^/Ecad^+^, and CD90^+^/Ecad^+^ cells, they may share similarities to the CD90^−^/Ecad^+^ cells.

**Figure 3:**
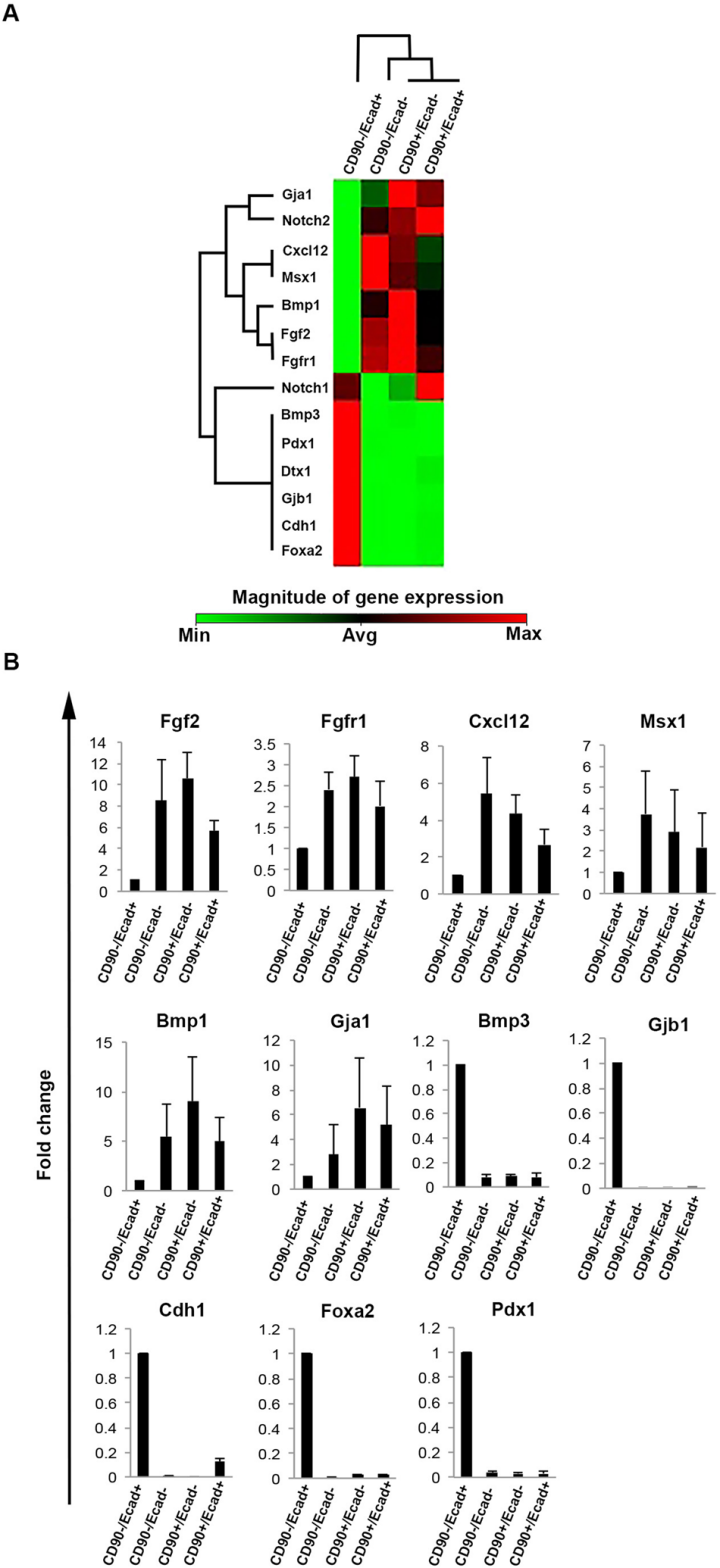
Gene expression analysis of different Aldefluor^+^ cell populations. (**A**) Hierarchical clustering of gene expression data among the cells analyzed in this study cluster CD90^−^/Ecad^−^, CD90^+^/Ecad^−^, and CD90^+^/Ecad^+^ populations together. (**B**) Expression of mesodermal, endodermal or pancreatic markers in four Aldefluor^+^ sub-populations. Fold change is the normalized gene expression in CD90^−^/Ecad^−^, CD90^+^/Ecad^−^, and CD90^+^/Ecad^+^ samples divided the normalized gene expression in the CD90^−^/Ecad^+^ sample. Cells in CD90^−^/Ecad^+^ sub-group displayed a strong endodermal profile and expressed Pdx1, whereas the other three sub-populations showed a more mesenchymal signature. Results are expressed as mean ± standard error of the mean (SEM). p < 0.05, (n = 3, where each sample was pooled cells from 3–5 mice).

To better discern the four Aldefluor^+^ populations, we used a targeted qPCR array for 84 genes related to the identification, growth and differentiation of stem cells, as well as specific markers for stem cells and pathways involved in maintenance of these cells, and found that the CD90^−^/Ecad^−^, CD90^−^/Ecad^+^, and CD90^+^/Ecad^+^ cell populations clustered together (Supplementary Fig. 6A). We next selected the genes that exhibited statistically significant differential expression in the CD90^−^/Ecad^−^, CD90^−^/Ecad^+^, and CD90^+^/Ecad^+^ cell populations compared to CD90^−^/Ecad^+^ cells (Fig. 3, Supplementary Fig. 6B). Interestingly, the expression of mesodermal markers such as Fgf2, Msx1, and BMP1 as well as the endodermal markers Fgfr1 and Cxcl12 was significantly higher in these cells compared to CD90^−^/Ecad^+^ cells. In contrast, CD90^−^/Ecad^+^ cells expressed the endodermal marker Foxa2, and the pancreatic marker Pdx1. Not surprisingly, Ecadherin (Cdh1) could be detected in both CD90^−^/Ecad^+^and CD90^+^/Ecad^+^cells, whereas it was absent in CD90^−^/Ecad^−^ and CD90^+^/Ecad^−^ populations. Furthermore, the Notch pathway appeared to be active in all four populations, indicating that regardless of their phenotype they may remain undifferentiated. The gene expression analysis confirms the mesenchymal nature of CD90^−^/Ecad^−^, CD90^−^/Ecad^+^, and CD90^+^/Ecad^+^ cells. Moreover, it implies that CD90^−^/Ecad^+^ cells may belong to a separate lineage than the other three Aldefluor^+^ populations. Interestingly, when we analyzed the E10.5 dorsal pancreatic mesenchyme, we found a high abundance of CD90^−^/Ecad^−^ cells in the examined tissue (Supplementary Fig. 7). The mesenchymal expression profile of CD90^−^/Ecad^−^ cells and the presence of similar cells in the dorsal mesenchyme, suggest that the progenitors to the CD90^+^/Ecad^−^ cells may originate in the mesenchyme surrounding the early embryonic pancreatic anlagen.

### Proliferation accounts for the expansion of Aldefluor^+^ cells

**Figure 4:**
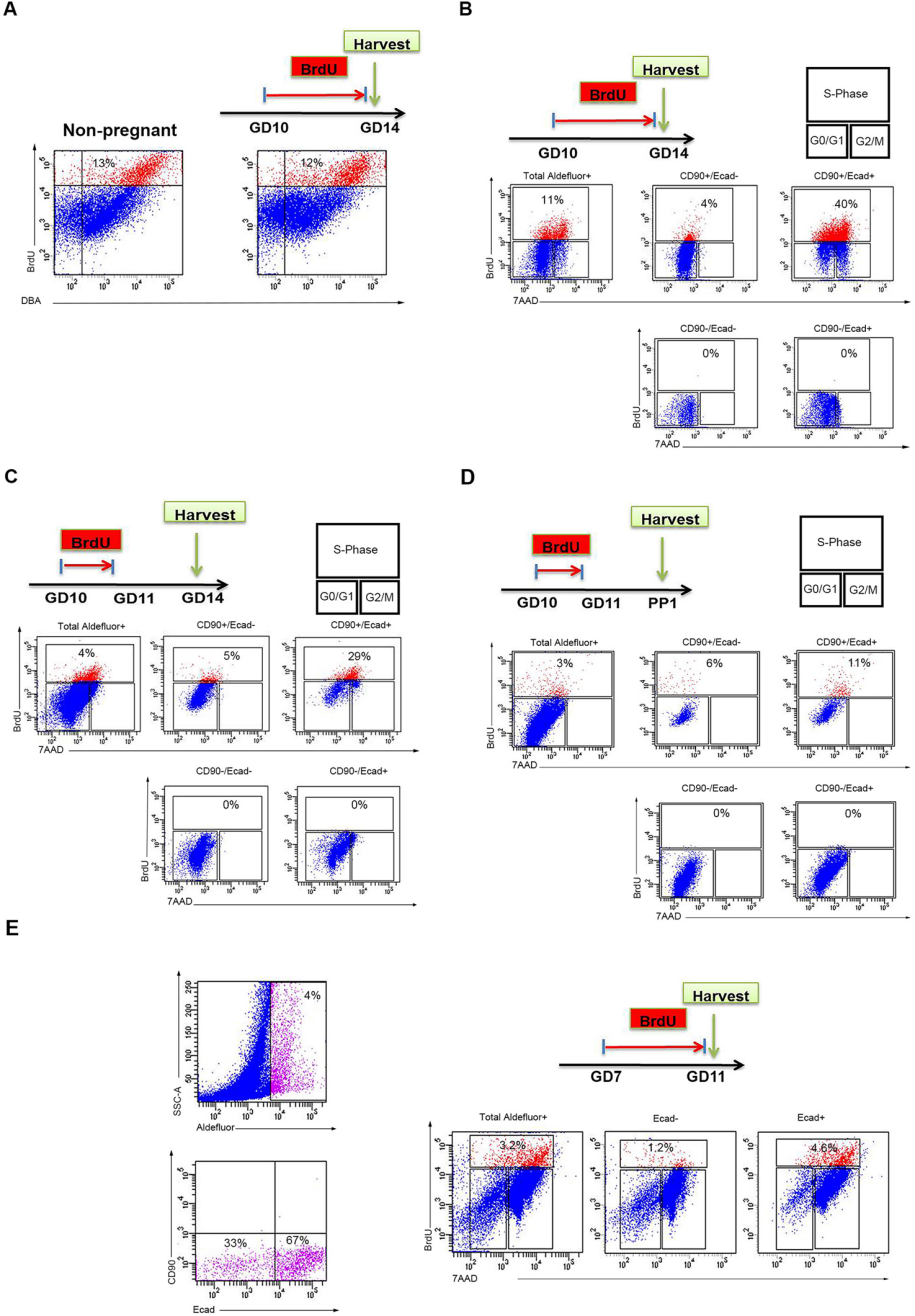
Proliferation analysis of the Aldefluor^+^ cells. (**A**) Flow cytometric analysis of BrdU incorporation during four days in DBA^+^ duct cells isolated from the pancreas of non-pregnant or aged-matched pregnant dams showing that duct cell proliferation is not influenced by pregnancy. (**B**) BrdU incorporation between gestational days 10 to 14 (harvest GD14) among the total Aldefluor^+^ population as well as within CD90^+^/Ecad^−^ or CD90^+^/Ecad^+^ sub-populations. (**C**) Pulse-chase BrdU treatment on gestational days 10 and 11 (harvest GD14) among the total Aldefluor^+^ population as well as within CD90^+^/Ecad^−^ or CD90^+^/Ecad^+^ sub-populations. (**D**) Pulse-chase BrdU treatment on gestational days 10 and 11 (harvest PP1) among the total Aldefluor^+^ population as well as within CD90^+^/Ecad^−^ or CD90^+^/Ecad^+^ sub-populations. Proliferation analyses indicate that the CD90^+^/Ecad^−^ cells are label retaining cells, whereas CD90^+^/Ecad^+^ cells are highly proliferative transient amplifying cells. Note the absence of BrdU incorporation in CD90^−^/Ecad^−^ or CD90^−^/Ecad^+^ cells in (**B**–**D**). (**E**) BrdU incorporation between gestational days 7 to 11 (harvest GD11) among the total Aldefluor^+^ population as well as within CD90^−^/Ecad^−^ or CD90^−^/Ecad^+^ sub-populations. Note that prior to the emergence of CD90^+^ cells, a subset of CD90^−^/Ecad^−^ and CD90^−^/Ecad^+^ cells proliferate. (**A**–**E**) are Pooled samples from 3 mice.

The expansion or emergence of different Aldefluor^+^ cell populations during pregnancy would require either influx of such cells from outside the pancreas, or else substantial proliferation and alterations of pre-existing pancreatic Aldefluor^+^ cells. To assess for the latter, BrdU was administered via the drinking water for four days, between gestational days 10 and 14 (Fig. 4A,B), after which the mice were sacrificed and the Aldefluor^+^ cells were stained for Ecadherin, CD90 and BrdU. In both pregnant and age-matched non-pregnant control females, BrdU-staining was seen in 12–13% of Aldefluor^−^/DBA^+^ cells, indicating that pregnancy does not stimulate proliferation of normal duct cells (Fig. 4A). During gestational days 10–14 around 11% of Aldefluor^+^ cells had incorporated BrdU (Fig. 4B). Next, we studied the distribution of BrdU^+^ cells within each Aldefluor^+^ subpopulation, and found that 4% of CD90^+^/Ecad^−^ cells, but 40% of CD90^+^/Ecad^+^ cells were proliferating, whereas CD90^−^/Ecad^−^ or CD90^−^/Ecad^+^ cells did not proliferate (Fig. 4B). Moreover, the BrdU^−^ cells within CD90^−^/Ecad^−^ population were predominantly in G0/G1 phase, whereas the BrdU^−^ cells in CD90^+^/Ecad^+^ group consisted of cells in both G0/G1 as well as G2/M-phases (Fig. 4B). To determine the fate of cells that were proliferating between gestational days 10 and 14, we then used a “pulse-chase” approach wherein BrdU was administered only between gestational days 10–11 and mice were sacrificed on gestational day 14 (Fig. 4C), or on postpartum day 1 (Fig. 4D). Around 5–6% of CD90^+^/Ecad^−^ cells were BrdU^+^ at both harvest times, whereas the percentage of CD90^+^/Ecad^+^ cells had dropped to 29% and 11.5% at GD14 and PP1, respectively. The observed decline is presumably due to either continued proliferation, with dilution of BrdU, or else due to changes in the surface marker status of those cells. Thus, the CD90^+^/Ecad^+^ cells appear to be a key proliferative transient amplifying cell (TAC) population, while the less proliferative CD90^+^/Ecad^−^ cells may function as label retaining cells (LRC). These results show that proliferation of Aldefluor^+^ cells seem to be restricted to the CD90-expressing CD90^+^/Ecad^−^ and CD90^+^/Ecad^+^ cells. However, as demonstrated in Fig. 2D and Supplementary Fig. 3B, the number of Aldefluor^+^ cells increases even before the emergence of CD90^+^ cells after GD11. Thus, we analyzed BrdU incorporation in pregnant dams prior to the appearance of CD90^+^ cells, and found that in the absence of CD90-expressing cells 3% of total Aldefluor^+^ cells were BrdU^+^, among which 1% were CD90^−^/Ecad^−^ cells, and 5% CD90^−^/Ecad^+^ cells (Fig. 4E). Noteworthy, here the BrdU^−^ cells in both populations consist mainly of cells in G2/M-phase. Our results show that all four sub-populations share the ability to proliferate. Nevertheless, after the appearance of CD90^+^ cells at GD11 the proliferation is predominantly within the CD90^+^ cells.

### Presence of a hierarchical relationship between Aldefluor^+^ sub-populations

**Figure 5:**
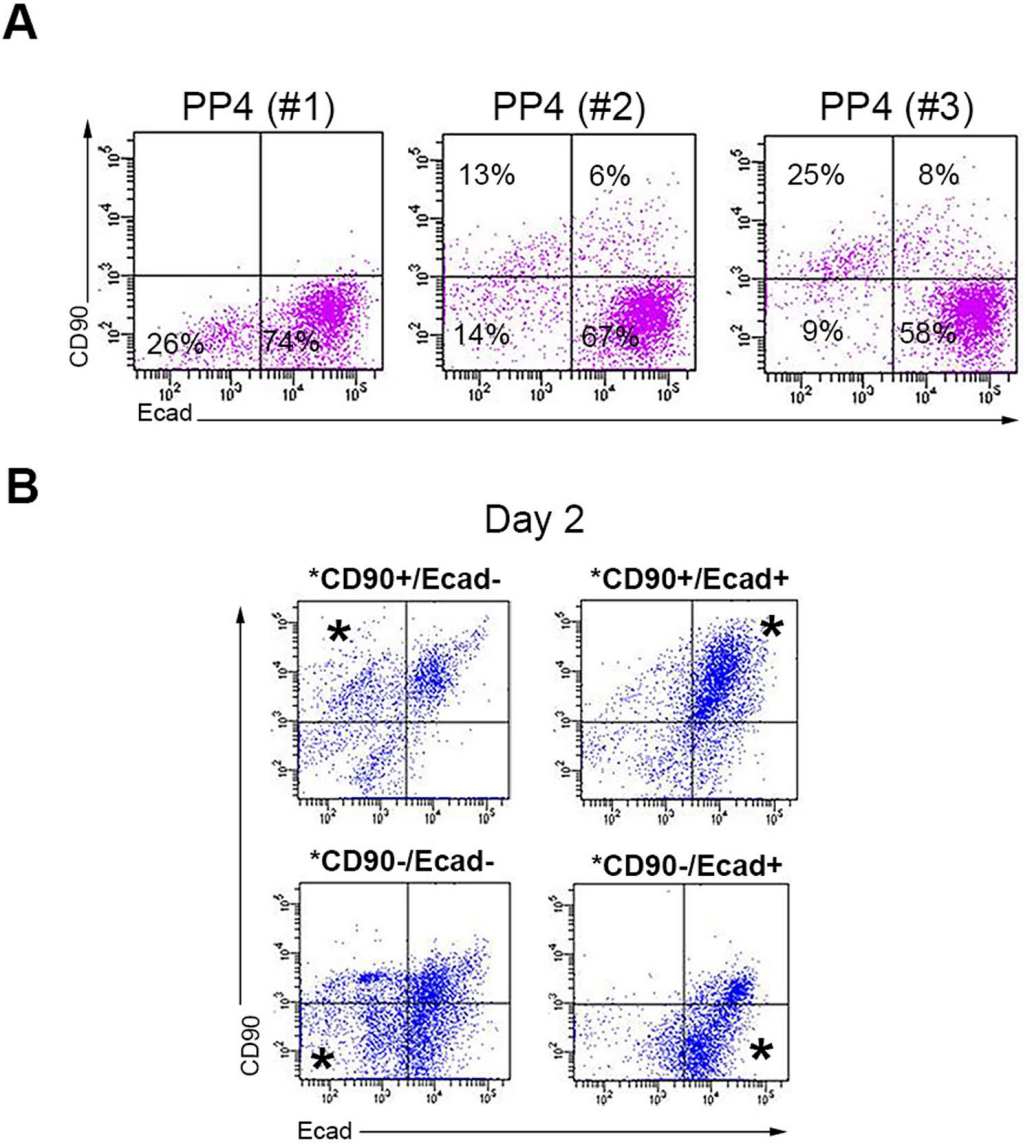
Hierarchical relationship between different sub-populations of Aldefluor^+^ cells. (**A**) Representative flow cytometric analysis of Ecadherin and CD90 expression in Aldefluor^+^ cells from the pancreas of three different postpartum day 4 dams. Note the inverse correlation between the presence of CD90^−^/Ecad^−^ cells compared with CD90^+^/Ecad^−^, and CD90^+^/Ecad^+^ cells. (**B**) The four Aldefluor sub-populations were isolated based on the Ecadherin and CD90 expression and cultured separately. Flow cytometric analysis of Ecadherin and CD90 expression in cells after 2 days in culture. Asterisks indicate the starting pool of cells enriched for one of the four sub-groups on day zero for each graph, respectively. Similar results were obtained in 3 independent studies. The corresponding numeric data for (**A**) is presented in Supplementary Fig. 4.

Interestingly, the emergence of the CD90^+^/Ecad^−^ and CD90^+^/Ecad^+^ sub-populations correlates with a decline in the number of CD90^−^/Ecad^−^ cells (Fig. 5A). Thus, we hypothesized that the CD90^−^/Ecad^−^ cells could represent progenitor cells that then can sequentially give rise to CD90^+^/Ecad^−^ cells (LRCs) and CD90^+^/Ecad^+^ cells (TACs) under certain conditions. To confirm this progenitor-progeny relationship, Aldefluor^+^ cells from postpartum day 1 dams were sorted based on CD90 and Ecadherin expression, and cultured separately (Fig. 5B). After two days in culture, CD90^−^/Ecad^−^ cells rapidly changed into cells in the other 3 quadrants. Noteworthy, a subset of CD90^+^/Ecad^−^ cells (LRC) remained CD90^+^/Ecad^−^, whereas the remaining became CD90^+^/Ecad^+^. Finally, CD90^+^/Ecad^+^ cells and CD90^−^/Ecad^+^ cells had maintained their original phenotype. Therefore, the CD90^+^/Ecad^+^ TACs are likely the final common pathway for the progression of more primitive cells from CD90^−^/Ecad^−^ and CD90^+^/Ecad^−^ subpopulations.

### Existence of adult cells that can commit to the pancreatic lineage *in vitro* and *in vivo*

**Figure 6:**
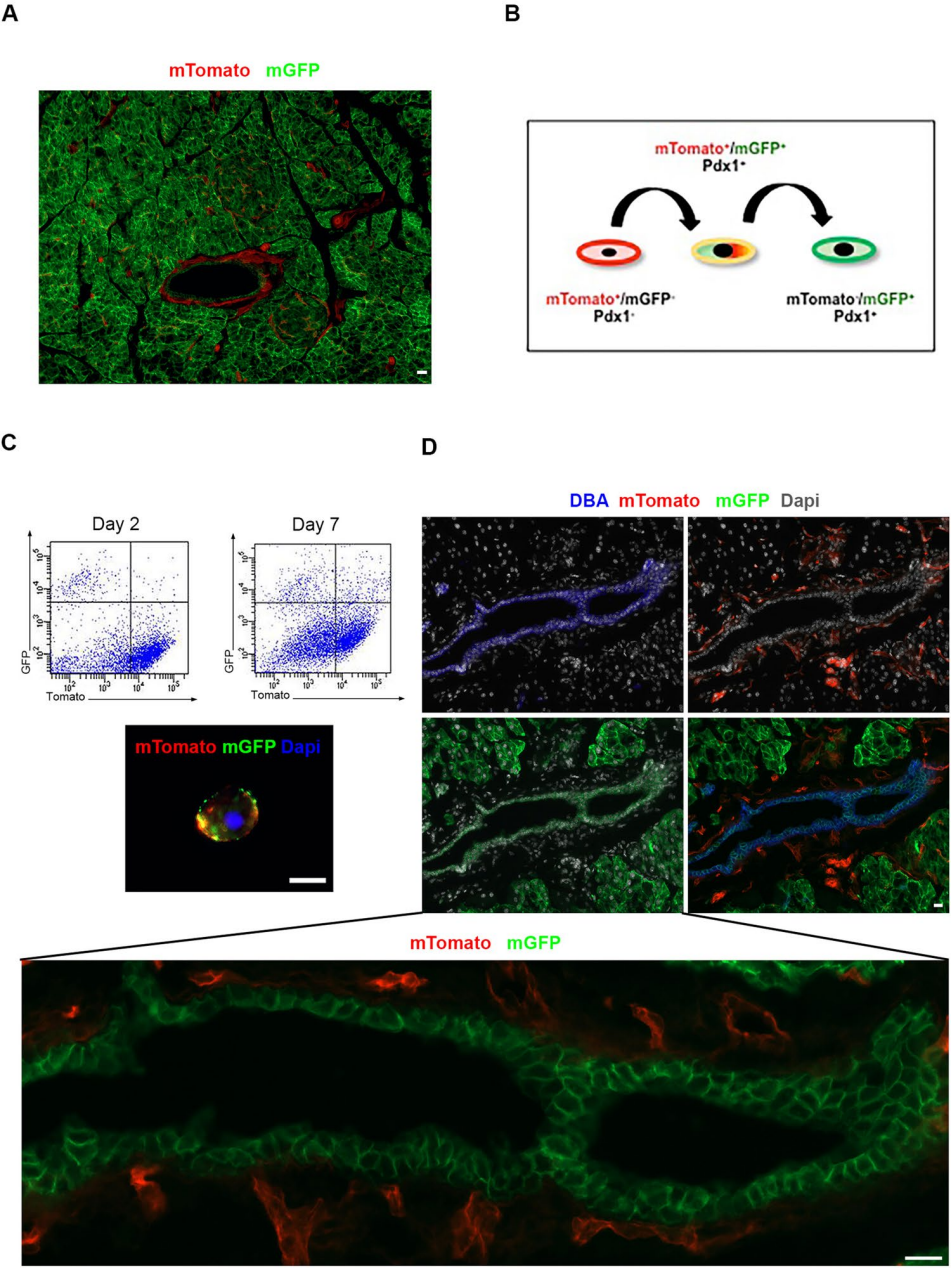
Near complete penetration of the Cre-recombinase in PdxCre;R26^*DTR/mTmG*^ mice. (**A**) Pancreatic tissues obtained from the PdxCre;R26^DTR/mTmG^ mice with no diphtheria toxin treatment were analyzed for mTomato and mGFP expression. (**B**) Schematic of PdxCre;R26^mTmG^ model as described in the text. (**C**) The Aldefluor^+^/CD90^−^/Ecad^-^ cells isolated from the pancreas of postpartum day 1 PdxCre;R26^*mTmG*^ dams were cultured and harvested after 2 or 7 days in culture. FACS analyses for mTomato and mGFP expression as well as fluorescent microscopy revealed the presence of mTomato^+^/mGFP^+^ “yellow” cells on day 7, indicating that these cells had recently and for the first time expressed Pdx1. Similar results were obtained in 5 independent studies. (**D**) Pancreatic tissues obtained from the PdxCre;R26^DTR/mTmG^ mice with no diphtheria toxin treatment were stained for the ductal marker DBA. Note that mTomato^+^ and mGFP^+^ cells are two separate populations in non-DT-treated mice (**A** and **D**). Bars 20 μm.

Our *in vitro* studies along with the expression analyses suggested that CD90^−^/Ecad^−^ cells are adult pancreatic multipotent cells, which could derive either from already committed but undifferentiated pancreatic cells through epithelial to mesenchymal transition, or alternatively may derive from a non-pancreatic pool of cells, perhaps originating from the pancreatic mesenchyme. The latter would imply that these cells must then express Pdx1 for the first time during the transition to a pancreatic cell fate. To distinguish between these two possibilities, we generated PdxCre;R26^*mTmG*^ compound mice by crossing PdxCre with R26^*mTmG*^ reporter mice. In these mice, all cells are mTomato^+^ except Pdx1-expressing cells and their progeny, in which, due to Cre-recombinase activity, the mTomato gene is excised and mGFP is instead expressed (Fig. 6A). If any cell would activate Pdx1-promoter for the first time, there would be a brief time window during which the cells are still mTomato^+^, but have begun to express mGFP, making them appear as having a yellow cell membrane (Fig. 6B). A similar approach was previously used to identify newly formed insulin-producing β-cells^33^.

Pregnant PdxCre;R26^mTmG^ dams were allowed to deliver and were sacrificed on postpartum day 1. Aldefluor^+^ cells were then gated for Ecadherin and CD90 expression, after which the Aldefluor^+^/CD90^−^/Ecad^−^ cells were FACS-sorted and cultured for a week (Fig. 6C). It should be emphasized that while it is not feasible to separate the Aldefluor^+^ cells from the mGFP^+^ pancreatic epithelial cells, the Aldefluor^+^ cells can easily be separated from the non-epithelial mGFP^−^ cells. The isolated cells were mainly mTomato^+^ at the time of harvest, and remained mTomato^+^/mGFP^−^ on day 2 (Fig. 6C), indicating they had not ever expressed Pdx1, perhaps arising from the embryonic pancreatic mesenchyme, which is PDX1^−^. However, by day 7 Tomato^+^ cells expressing mGFP could be detected, indicating the expression of Pdx1 for the first time (Fig. 6C). The fact that the cultured cells were sorted from the Aldefluor^+^/Ecad^−^ population makes it unlikely that these are contaminating pancreatic epithelial cells that might have escaped Cre-recombinase activity. Furthermore, membrane localization of GFP, and the presence of only one nuclei in the mTomato^+^/mGFP^+^ cells on day 7 (Fig. 6C) would argue against the possibility of fusion between mTomato^+^ and mGFP^+^ cells.

**Figure 7:**
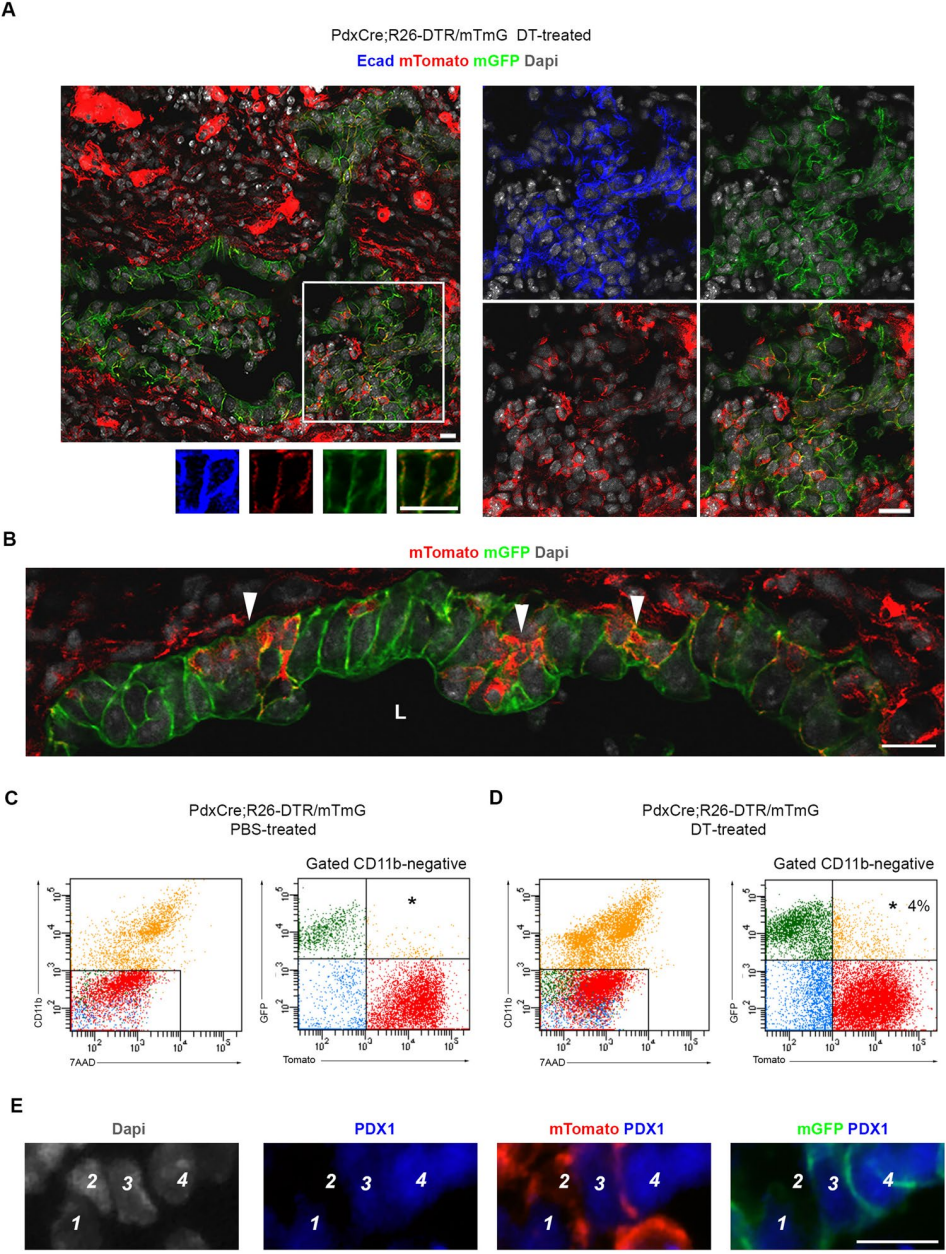
A subset of cells with ALDH-activity in the adult pancreas can commit to the pancreatic program for the first time. (**A**,**B**) Confocal imaging of pancreatic tissues obtained from the DT-treated PdxCre;R26^DTR/mTmG^ mice where mTomato^+^/mGFP^+^ cells (yellow cells) could be observed within the regenerative ductal structures. Right and lower panels in (**A**) are higher magnification of the highlighted areas in the left panel. Arrowheads in (**B**) indicate foci of mTomato^+^/mGFP^+^ cells (yellow cells) along the regenerative ducts. Similar results were obtained in 3 out of 3 DT-treated PdxCre;R26^DTR/mTmG^ mice. (**C**,**D**) Total pancreatic cells isolated from PdxCre;R26^DTR/mTmG^ mice treated with PBS (**C**), or DT (**D**) were first stained for CD11b (left graphs in each figure). The CD11b^−^ gated cells (marked with rectangle) were analyzed for mTomato and mGFP (right graphs in each figure). (**E**) Tissues obtained from the DT-treated PdxCre;R26^DTR/mTmG^ mice were stained for PDX1. Pdx1-expression was absent in the Tomato^+^/GFP^−^ cell (2), whereas the Tomato^−^/GFP^+^ cell (4) displayed high level of Pdx1. Note the low expression of Pdx1 in the Tomato^+^/GFP^+^ cells (1 & 3). L: lumen. Bars 20 μm.

In a previous study, we showed that following an extensive diphtheria toxin-induced (DT) ablation of nearly all acinar and endocrine cells (PdxCre;R26^*DTR*^ mice), surviving cells within the ductal network are capable of contributing to both endocrine and acinar regeneration via recapitulation of the embryonic pancreatic developmental program^8^ (Supplementary Fig. 8A). Based on the ability of a subset of Aldefluor^+^ cells to turn on Pdx1 expression *in vitro* (Fig. 6C), and the significant expansion of Aldefluor^+^ cells (both Ecadherin^+^ and Ecadherin^−^) in the PdxCre;R26^*DTR*^ mice as the result of DT-treatment (Supplementary Fig. 8B), we generated PdxCre;R26^*DTR/mTmG*^ mice to explore the possibility to find cells that recently have committed to the pancreatic fate *in vivo*. As demonstrated in Fig. 6D, the pancreas of non-DT treated control PdxCre;R26^*DTR/mTmG*^ mice consisted of mTomato^−^/mGFP^+^ pancreatic cells, or mTomato^+^/mGFP^−^ non-pancreatic (mainly endothelial) cells (Fig. 6D). Moreover, we could not find any mTomato^+^/mGFP^−^ cells within the ductal compartment (Fig. 6D). On the other hand, in DT-treated PdxCre;R26^*DTR/mTmG*^ mice we were able to detect mTomato^+^/mGFP^+^ scattered within the regenerative ductal compartment (Fig. 7A). Of note, the mTomato^+^/mGFP^+^ cells were not present randomly within the ducts. Instead, we could detect clusters of mTomato^+^/mGFP^+^ cells appearing in intervals along the regenerative ducts (Fig. 7B). To further confirm the existence of these mTomato^+^/mGFP^+^ cells, we next isolated cells from the pancreas of non-DT treated (Fig. 7C) or DT-treated (Fig. 7D) PdxCre;R26^*DTR/mTmG*^ mice. Following DT/DTR-mediated cell ablation, the injured pancreas is infiltrated by the macrophages^30^. In DT-treated PdxCre;R26^*DTR/mTmG*^ mice, macrophages (mTomato^+^) engulf the apoptotic pancreatic cells (mGFP^+^) and therefore may appear “yellow”. In order to avoid these mTomato^+^/GFP^+^ macrophages, we first stained the cells for the macrophage marker CD11b, and then analyzed the gated CD11b-negative population for mTomato and mGFP expression. Our FACS analyses showed no mTomato^+^/mGFP^+^ cells in the non-DT treated PdxCre;R26^*DTR/mTmG*^ pancreas (Fig. 7C), whereas DT-treatment resulted in the appearance of mTomato^+^/mGFP^+^ cells (Fig. 7D). In the PdxCre;R26^*DTR/mTmG*^ mice, the Cre-recombinase expression is under the control of an exogenous Pdx1 promoter. To determine whether the observed Cre-recombinase activity and the subsequent mTomato-to-mGFP switch is associated with de novo Pdx1 expression, we immune-stained the DT treated PdxCre;R26^*DTR/mTmG*^ pancreas and found that the mTomato^+^/mGFP^+^ cells are indeed PDX1^+^ (Fig. 7E). Noteworthy, the mTomato^+^/mGFP^+^ cells displayed lower PDX1 levels than the adjacent mTomato^-^/mGFP^+^ cell, indicating a recent onset of Pdx1 expression in those cells. The mTomato^+^/mGFP^+^ cells expressed Ecadherin, and were not endothelial cells (Supplementary Fig. 8C). More importantly, the fact that these mTomato^+^/mGFP^+^/Ecad^+^ cells did not express the macrophage marker F4/80 (Supplementary Fig. 8D) argues against the possibility that these are apoptotic pancreatic cells being engulfed by macrophages. Finally, the absence of mTomato^+^/mGFP^−^ ductal cells would exclude the possibility that escaper duct cells, which failed to undergo Cre-recombination during development, would be newly-labeled as the result of upregulated Pdx1 expression following DT-treatment^8,30^.

Together, our *in vitro* and *in vivo* data suggest that, in the adult pancreas progeny of the Aldefluor^+^/CD29^+^/CD90^−^/Ecad^−^ cells may have the ability to commit to pancreatic cell lineage.

## Discussion

Our *in vivo* and *in vitro* analyses of cells with high ALDH-activity have yielded some surprising findings. First, these multipotent cells display non-epithelial characteristics and may originate from the mesenchyme surrounding the embryonic pancreatic anlagen. Second, they have the ability to express Pdx1 when provided the proper signals.

We have found significant expansion of Aldefluor^+^ cells during post-natal growth, pregnancy, and following pancreatic injury. The ALDH superfamily consists of many members, among which Aldh1a1, Aldh1a2, and Aldh1b1 are expressed in the embryonic and/or in the adult mouse pancreas^29,36–38^. ALDH catalytic activity, determined by Aldefluor assay does not discriminate between different ALDH proteins. While the potential involvement of these cells in the post-natal growth of the pancreas is unknown, our lineage tracing studies indicate that at least Aldh1a1-expressing cells and their progeny participate in maintenance of the acinar compartment in the adult mouse pancreas. Furthermore, these cells in response to physiological demands associated with pregnancy and lactation, can also differentiate into β-cells. It has been reported that during gestational days 9 and 12 in mice, proliferating β-cells display ALDH activity as evidenced by the emergence of Aldefluor^+^/insulin^+^ cells^39^. Therefore, it is possible that the Aldh1a1 lineage-tagged β-cells in our study are these proliferating Aldefluor^+^/insulin^+^ cells. However, since we could detect tomato-labeled β-cells first on postpartum day 14 and onward, it is unlikely that the Aldh1a1 lineage-tagged β-cells reported here are β-cells that transiently express Aldh1a1 between GD9-GD12.

Cells with ALDH-activity in the pancreas were originally reported to be either Ecadherin^+^ or Ecadherin^−^^29^. Here, we show that these two populations can be further divided based on CD29 and CD90 expression. The CD29^+^/CD90^−^/Ecad^−^ cells clearly display a mesenchymal signature, and are rare in baseline conditions, but can proliferate and give rise to the CD29^+^/CD90^+^/Ecad^−^ cells. Intriguingly, we found Aldh1a1 lineage-labeled cells outside the parenchyma in an area which is typically associated with the mesothelial lining of the pancreas^40^. Further studies are required to reveal the true identity of these cells. Nevertheless, prior to differentiating into pancreatic cell types, the aforementioned non-epithelial cells or their progenies would have to acquire epithelial features. Weinberg and colleagues have shown that human mammary epithelial cells acquire stem-like features by expressing CD90 during epithelial-to-mesenchymal transition^34^. Our data suggest that in order to gain stem-like properties, CD29^+^ pancreatic Ecad^−^ cells similarly express CD90, but instead initiate a mesenchymal to epithelial transition process. The fourth population consisted of CD29^+^/CD90^−^/Ecad^+^ cells, which were previously reported by Leach and colleagues to be present in both the embryonic and the adult pancreas^29^. The CD90^−^/Ecad^+^ cells also appeared to be the most heterogeneous group among the four Aldefluor^+^ populations. Because (i) CD90^−^/Ecad^+^ cells express Pdx1 throughout development^29^, (ii) their expression profile does not cluster them with the other Aldefluor^+^ populations, and (iii) their expansion and decline during pregnancy and lactation do not show any clear correlation with the other three cell types, it is likely that the CD90^−^/Ecad^+^ cells derive from a separate lineage than the other three subpopulations.

The pancreas has a low rate of spontaneous self-renewal, and as pancreatic regeneration relies mainly on the proliferation of differentiated cells^4,41,42^, actively proliferating stem cells would seem superfluous. On the other hand, a mechanism that maintains a stem cell population in a quiescent state, only proliferating when needed, seems more efficient. Of note, the CD90^+^/Ecad^−^ cells demonstrate a label retaining capacity, as 4–5% of the cells in this population incorporated BrdU regardless of the window of BrdU administration, whereas a subset of CD29^+^/CD90^+^/Ecad^+^ cells display a highly proliferative capacity. Together, these data indicate that the CD90^+^/Ecad^−^ cells may represent a dormant stem cell population, which gives rise to the more proliferative transient amplifying CD90^+^/Ecad^+^ cells. To our knowledge our findings are the first demonstration of quiescent and active stem cells coexisting in the adult pancreas.

The exact origin of Aldefluor^+^/CD29^+^/CD90^−^/Ecad^−^ cells remains unknown. However, since cells with similar expression profile can be found in the E10.5 dorsal mesenchyme, it is tempting to speculate that the CD90^−^/Ecad^−^ cells that we found in the adult pancreas may originate from Aldefluor^+^/CD29^+^/CD90^−^/Ecad^−^ cells in the dorsal pancreatic mesenchyme. Regardless of their origin, the progeny of these Aldefluor^+^/CD29^+^/CD90^−^/Ecad^−^ cells seem to have the ability to turn on Pdx1 expression when needed to initiate a pancreatic lineage-specific program. Interestingly, these cells express Fgf2, along with endodermal markers Fgfr1 and Cxcl12, which are all involved in early specification of the pancreatic domain^43–45^. FGF2 is also known to be required for mesenchymal condensation during MET^1^. Thus, it is noteworthy that among the four sub-populations, the Aldefluor^+^/CD29^+^/CD90^+^/Ecad^−^ cells displayed the highest levels of Fgf2-expression.

Using the R26^mTmG^ reporter line enabled us to show that progeny of Aldefluor^+^/CD29^+^/CD90^−^/Ecad^−^ cells can acquire a pancreatic fate. A limitation with the R26^mTmG^ is that the mTomato^+^/mGFP^+^ cells do not remain permanently yellow. Thus, as the yellow cells lose mTomato and gain mGFP in the DT-treated PdxCre;R26^*DTR/mTmG*^ mice, they become indistinguishable from the rest of the mGFP^+^ PDX1-lineage. The transient co-existence of mTomato and mGFP makes any attempts to quantify the number of newly committed cells or the differentiated cells deriving from these cells in the regenerated pancreas impractical. We have shown that in the adult PdxCre;R26^*DTR*^ mice, DTR is expressed by all Pdx1-progenies^8^. Surprisingly, DT-induced ablation is seen specifically in the acinar and the endocrine lineages, whereas the ductal cells survive^8^. Thus, in that study while we were not able to identify the specific cells that could serve as the cell of origin in the robust pancreatic regeneration seen in the DT/DTR-mediated injury model, we proposed that surviving cells within the ductal network might have contributed to the reported regenerative process. Identification of the cells that had recently committed to the pancreatic program embedded within the regenerative ducts in the DT-treated PdxCre;R26^*DTR/mTmG*^ mice makes these cells a potential candidate as cell of origin. However, future studies will have to explore the true regenerative capacity of these cells. Nevertheless, the ability to express Pdx1 for the first time is a novel finding and may explain why regenerated pancreatic cells were not lineage-tagged by typical duct markers in previous studies^12,13^.

**Figure 8:**
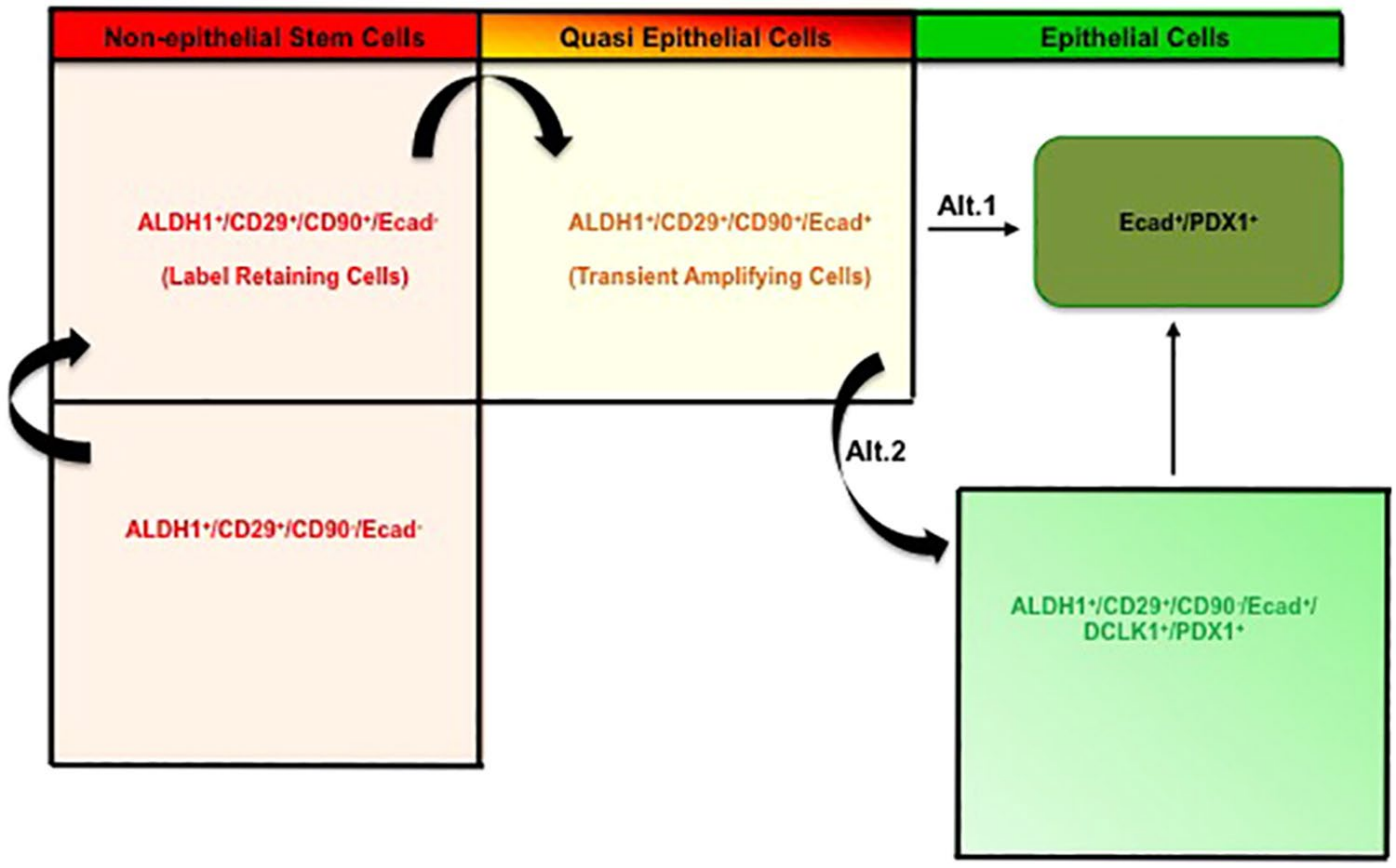
Model of the hierarchy between the different sub-populations of cells with ALDH-activity, as described in the text.

In conclusion, the Aldefluor^+^ cells expand during and after pregnancy, or in pathological conditions (Fig. 8). The ALDH1^+^/CD29^+^/CD90^−^/Ecad^−^ cells initially expand, express CD90 and become the ALDH1^+^/CD29^+^/CD90^+^/Ecad^−^ cells (LRC), followed by conversion to Aldefluor^+^/CD29^+^/CD90^+^/Ecad^+^ cells (TAC). Finally, these cells can then commit to a pancreatic program by expressing Pdx1 for the first time in conjunction with loss of ALDH activity (alternative 1), or remain in a quiescent, progenitor-like state with expression of DCLK1^+^ (alternative 2). The cells presented in this study may have the potential to be used for therapeutic purposes, as they are endogenous sortable cells present in diabetic and pancreatitis patients.

## Methods

### Human tissue specimens

Human pancreatic specimens were collected from deceased organ donors or from tissues otherwise discarded from pancreatitis patients undergoing total pancreatectomy for autologous islet transplantation. All studies with human pancreatic tissues were approved by the University of Pittsburgh Institutional Review Board. All surgically removed pancreatic tissues were collected after written patient consent and in accordance with the University of Pittsburgh Institutional Review Board-approved protocols.

### Mice

Mice used in these studies were maintained according to protocols approved by the University of Pittsburgh Institutional Animal Care and Use Committee. The Rosa26^*CAGTomato*^ ^46^, Rosa26^*mTmG*^ ^47^, Rosa26^*DTR*^ ^8,30^, wild type C57bl/6 and NOD mice were purchased from The Jackson Laboratory (Bar Harbor, ME). The PdxCre^8,48^ mice were obtained from the Mouse Models of Human Cancer Consortium. We have observed that approximately 25% of the PdxCre;Rosa26^*DTR*/*mTmG*^ mice display low Cre-recombinase penetrance. In this study, we have excluded the mice with low Cre-penetrance and used transgenic mice with near complete recombination. Wild type CD-1 mice were purchased from the Charles River Laboratories.

### Isolation and culture of Aldefluor^+^ cells

Total mouse pancreas or non-islet fraction of human pancreas were used for isolation of Aldefluor^+^ cells. For adult time points (postnatal age > P14) pancreata were digested and analyzed singularly. For early postnatal time points (<P14) due to their small size, a variable number between 7–10 organs was pooled together and considered as one replicate. After harvesting, pancreata were finely minced and put into a 50 ml conical tube in a solution containing 2 mg/ml Collagenase type V (Sigma-Aldrich) in HBSS with Ca^2+^ and Mg^2+^. Digestion was carried out in a shaking water bath at 37 °C for 8–10 minutes and stopped with ice-cold HBSS without Ca^2+^ and Mg^2+^. The digested tissue was subsequently filtered through a 40 μm pre-wet cell strainer in order to remove large acinar clumps and islets. Cells were then centrifuged for 5 minutes at 1300 rpm and filtered into polystyrene round bottom tubes with 35 μm cell strainer caps. If necessary, red cell lysis was performed (Red Blood Cell Lysis Buffer Hybri-Max, Sigma-Aldrich) according to the manufacturer’s instructions. For surface staining, cells were then washed, counted, resuspended at 10^7^ cells/100 µl of Separation/Sorting Buffer (PBS without Ca^2+^, 0.5% BSA and 2 mM EDTA) and incubated with primary antibodies for 20 minutes at 4 °C in the dark. After washing, the cells were incubated with secondary antibodies for 20 minutes at 4 °C in the dark. Finally, the cells were washed and resuspended in 300–500 μl of sorting buffer to be analyzed by flow cytometry, or used for further assays.

The aldehyde dehydrogenase activity was detected using the Aldefluor assay kit (StemCell Technologies) as described by the manufacturer’s instructions. Briefly, dissociated single cells were resuspended in Aldefluor assay buffer containing an ALDH substrate, bodipy-aminoacetaldehyde (BAAA), and immediately incubated for 35 minutes at 37 °C. A specific inhibitor of ALDH, diethylaminobenzaldehyde (DEAB) was used as negative control. For cell culture purpose, Aldefluor^+^ cells were sorted with a BD FACSAria Cell Sorter, washed twice in PBS without Ca^2+^, 0.5% BSA and 2 mM EDTA, centrifuged for 5 minutes at 1300 rpm and cultured in a humidified incubator with 5% CO_2_ at 37 ^o^C, in 24 well ultra-low attachment plates (Costar) or in chamber slides (Thermo Fisher), for 2 to 7 days in a serum-free media containing DMEM/F12 + GlutaMAX (Gibco) supplemented with 1x B27 (Invitrogen), 20 ng/mL bFGF (Invitrogen), 20 ng/mL EGF (Invitrogen), 100 UI/ml penicillin and 0.1 mg/ml streptomycin (Corning, NY)^49^. The cells were fed every 3 days with media containing growth factors (bFGF and EGF). After 2 to 7 days in culture, cells were fixed with 4% PFA, stained with Dapi and visualized under a fluorescent microscope (Carl Zeiss Axio Imager Z1), or stained with primary and secondary antibodies following the procedure mentioned above, before subjected to FACS analysis.

### Flow Cytometry analysis

For surface staining, cells were incubated with primary conjugated antibodies for 20 minutes at 4 °C in the dark. For intracellular cytokine staining, BD Cytofix/Cytoperm Fixation/Permeabilization solution Kit (BD Bioscience, Cat. No. 554714) was used according to the manufacturer’s instructions. Cells were then incubated with conjugated intracellular antibodies for 20 minutes at 4 °C in the dark. Data were acquired with a BD FACSAria Cell Sorter, analyzed with FACSDiva Software (BD Bioscience). For BrdU detection, Aldefluor positive cells were sorted by FACS, fixed for 15 minutes at 4 °C with Cytofix and incubated with a BrdU antibody conjugated with BV510, for 20 minutes at 4 °C. Cells were then washed with perm wash, stained with 5 μl of 7-aminoactinomycin D (7AAD) (BD Pharmingen) per test for 10 minutes at 4 °C, and immediately analyzed by flow cytometry.

### Immunfluorescence Analysis

Tissue and whole mount processing, Immunostaining, and quantification analysis were performed as previously described^8,50^. The antibodies used in this study are listed in Supplementary Table 1.

### Gene expression analysis – PCR array

Total RNA was isolated from all different cell populations using an RNA extraction kit according to the manufacturer’s instructions (RNeasy Mini Kit, QIAGEN, Cat. no. 74104). RNA quality was determined using a spectrophotometer and was reverse transcribed using a cDNA conversion kit (RT² First Strand Kit, QIAGEN, Cat. no. 330401). The cDNA was used on the real-time RT² Profiler PCR Array (QIAGEN, Cat. no. PAMM-405Z) in combination with RT² SYBR® Green qPCR Mastermix (Cat. no. 330529). CT values were exported to an Excel file to create a table of CT values. This table was then uploaded on to the data analysis web portal at http://www.qiagen.com/geneglobe. Samples were assigned to controls (CD90^−^/Ecad^+^ cells) and test groups (CD90^−^/Ecad^−^, CD90^+^/Ecad^−^, and CD90^+^/Ecad^+^ cells). CT values were normalized based on a Manual Selection of reference genes. The calculation of fold change/regulation was done using delta delta CT method, in which delta CT is calculated between gene of interest and an average of reference genes (HKG), followed by delta-delta CT calculations (delta CT (Test Group)-delta CT (Control Group)). Fold Change is then calculated using 2 (−delta delta CT) formula. The p values were calculated based on a Student’s t-test of the replicate 2 (−Delta CT) values for each gene in the control group and test groups. The number of biological and technical replicates was 3, as recommended by the manufacturer.

### Diphtheria toxin, and caerulein treatment

PdxCre;R26^*DTR/mTmG*^ mice were treated intraperitoneally with 100ng of DT for 5 consecutive days as described previously^8,30^. Pancreatic acinar cell injury was induced by 8 time doses of 50 μg/kg caerulein, injected intraperitoneally 1 hour apart over 2 consecutive days, as described previously^42^.

### BrdU labeling

Mice were treated for 1 or 4 days with 0.8 mg BrdU/ml in drinking water.

### Quantitative Real-Time PCR

For qRT-PCR, messenger RNA isolation and subsequent complementary DNA synthesis were performed using μMACS One-step cDNA kit (Miltenyi Biotec, Cat. No. 130–091–902), according to the manufacturer’s instructions. Reactions were performed with PerfeCTa SYBR Green SuperMix for IQ (Quanta Biosciences, Cat. No. 95053) on a BioRad IQ5 Instrument (Biorad). Reactions were performed at least in triplicates and specificity of the amplified products was determined by melting peak analysis. Quantification for each gene of interest was performed with the 2^−ΔΔCt^ method. Quantified values were normalized against the housekeeping gene GAPDH. PCR primers were purchased from Qiagen (QuantiTect Primer Assays, Qiagen) are listed here. The primers used in this study are listed in Supplementary Table 2.

### AAV6 vector construction

The AAV-ALDH1A1-Cre vector was constructed by amplifying the Aldh1a1 promoter region by PCR and subcloning into the EcoRI and NheI sites of the vector pAAV-CMV-Cre^32^. The 2 kb promoter used in this construct has been shown to regulate Aldh1a1 gene expression^31^. The primers used were Aldh1a1 Forward EcorI: 5′-GAATTCAAATGGGCAGGCATGGTAAC-3′, and Aldh1a1 Reverse NheI: 5′-GCTAGCTGGCTCCTGGAACAC-3′. Recombinant adeno-associated virus vectors were produced and purified as previously described^51^. Packaging plasmids carrying the serotype rep and cap genes and helper plasmid carrying the adenovirus helper functions were purchased from Applied Viromics, LLC. (Fremont, CA).

### Gene delivery through ductal infusion

This system entails a special technique where a catheter is introduced tangentially across the wall of the duodenum, and then into the common pancreatic/bile duct and has been previously described in detail^32^.

### Statistical analysis

Statistical significance between groups was determined using a Student’s t-test. Most calculations were performed using GraphPad prism. *P* < 0.05 was considered as statistically significant. The number of independent biological replicates (n) for each experiment is indicated in the corresponding figure legends.

## Electronic supplementary material

Supplementary Information
