# Supplementary material for: Identification of Newly Committed Pancreatic Cells in the Adult Mouse Pancreas
Source: Sci Rep. 2017 Dec 13;7:17539. doi: 10.1038/s41598-017-17884-z (PMC5727523; doi:10.1038/s41598-017-17884-z)
Supplement: Supplementary file 1 — Supplementary Information [file 41598_2017_17884_MOESM1_ESM.pdf]

Identification of Newly Committed Pancreatic Cells in the Adult Mouse Pancreas

Mairobys Socorro[1,2,8], Angela Criscimanna[1,2,8], Patricia Riva[1,2], Manuj Tandon[1,2], Krishna Prasadan[1,2], Ping Guo[1,2,#], Abhinav Humar[1], Sohail Z. Husain[3], Steven D. Leach[4], George K. Gittes[1,2], and Farzad Esni[1,2,5,6,7,*]

[1] Department of Surgery, [2] Division of Pediatric General and Thoracic Surgery, Children's Hospital of Pittsburgh, University of Pittsburgh Medical Center, One Children's Drive, 4401 Penn Avenue, Rangos Research Center, Pittsburgh, PA 15244, USA.
[3] Department of Pediatrics, Children's Hospital of Pittsburgh, University of Pittsburgh Medical Center, Pittsburgh, PA 15244, USA.
[4] Rubenstein Center for Pancreatic Cancer Research, Memorial Sloan Kettering Cancer Center, New York, NY, 10065, USA.
[5] Department of Developmental Biology, University of Pittsburgh, Pittsburgh, PA 15244, USA.
[6] Department of Microbiology and Molecular Genetics, University of Pittsburgh, Pittsburgh, PA 15244, USA.
[7] University of Pittsburgh Cancer Institute, Pittsburgh, PA, 15123, USA.
[8] These authors contributed equally to this work

[#] Current address: Department of Orthopaedic Surgery, University of Texas Health Science Center at Houston,1881 East Road, 3SCR6.4621, Houston, Texas 77054

Correspondence and request for materials should be addressed to Farzad Esni (farzad.esni@chp.edu).

Supplementary Figure Legends

Supplementary Figure 1: Isolation of a pool of cells enriched for duct cells. (A) Flow cytometric analysis of unstained cells, Ecadherin alone, DBA alone, or Ecadherin and DBA combined. This cell population is enriched for duct cells from the adult pancreas according to the protocol described in the Methods. (B) Representative flow cytometric analysis of ALDH-activity (Aldefluor) in a similar pool of cells enriched for duct cells from the pancreas of adult mice (n=10). (C) qRTPCR for insulin or amylase expression in duct cells (DBA^+), $Aldefluor^+$ cells, or total pancreas (n=3, where each sample was pooled cells from 3-5 mice). (D, E) Representative flow cytometric analysis of ALDH-activity in cells from the pancreas of (D) post-natal mice (n=2, pooled samples from 7-10 pups for each time point), (E) pregnant (GD11 n=8, GD14 n=14, GD17 n=44) or postpartum dams (PP1 n=31, PP4 n=37, PP7 n=12, PP14 n=5, PP21 n=4). $Aldefluor^+$ cells are highly abundant before weaning in neonatal mice, as well as in pregnant and postpartum dams. $Aldefluor^+$ cells isolated from human pancreas display a similar expansion pattern to their mouse counterparts. P0-P21: post-natal days 0 to 21; GD11-GD17: gestational days 11 to 17; PP1-PP21: postpartum days 1 to 21; DBA: Dolichos Biflorus Agglutinin.

Supplementary Figure 2: Cells with ALDH-activity are abundant in the mouse pancreas. (A) Representative flow cytometric analysis of ALDH-activity (Aldefluor) in cells from the head, body or tail of the pancreas of pregnant mice (upper panel) showed equal distribution of $Aldefluor^+$ cells in different parts of the adult mouse pancreas. Flow cytometric analysis of Ecadherin expression in $Aldefluor^+$ cells from the head, body and tail of the pancreas of pregnant mice showed a similar ratio of $Aldefluor^+/Ecadherin^+$ cells throughout the gland (lower panel) (n=5). (B) Representative flow cytometric analysis of Ecadherin and ALDH-activity (Aldefluor) in cells from the pancreas of gestational day 17 pregnant (n=24), or postpartum days 14 (n=10) or 21 dams (n=12). (C)

Quantification of percentages of Ecad^{+} cells within the Aldefluor^{+} population in the pancreas of non-pregnant (n=7), pregnant (GD11 n=5, GD14 n=14, GD17 n=24) or postpartum dams (PP1 n=25, PP4 n=26, PP7 n=12, PP14 n=5, PP21 n=4). (D) Representative flow cytometric analysis of F4/80 and CD11b, or CD31 expression in Aldefluor^{+} cells from the pancreas of pregnant (GD17) dams, showing that Aldefluor^{+} cells isolated from the pancreas are not macrophages nor endothelial cells (n=5). (E) Representative flow cytometric analysis of Ecadherin expression in Aldefluor^{+} cells from the pancreas of postnatal day 7 pups (n=2, pooled samples from 7-10 pups), caerulein-treated (n=5) or diabetic NOD mice (n=4) showed expansion of both Ecadherin-positive as well as Ecadherin-negative cells in these conditions (n=5). (F) Representative flow cytometric analysis of Ecadherin expression in Aldefluor^{+} cells from the pancreas of healthy pediatric (n=3), chronic pancreatitis (n=3), or T1D (n=3) patients showed expansion of both Ecadherin-positive as well as Ecadherin-negative cells in human pancreas.

Supplementary Figure 3: Characterization of different Aldefluor^{+} cell populations. (A, B) Representative flow cytometric analysis of stem cell markers Sca-1, CD44, CD45, CD105, or CD133 expression, in Aldefluor^{+} cells from the pancreas of non-pregnant (A), or postpartum (B) dams showing that Aldefluor^{+} cells isolated from the mouse pancreas do not express the aforementioned stem cell markers (n=5).

Supplementary Figure 4: Aldefluor^{+} cells can be separated into four sub-populations. (A) Representative flow cytometric analysis of Ecadherin and CD90 expression in Aldefluor^{+} cells from the pancreas of gestational day 17 pregnant, postpartum days 14 and 21 dams. (B) Quantification of the actual number of the four Aldefluor^{+} sub-population in the pancreas of non-pregnant adult, pregnant, or postpartum dams at indicated stages. GD: Gestational Day; PP: Postpartum. Results are expressed as the mean ± SD. (C) Quantification of the percentage of the four Aldefluor^{+} sub-population in the pancreas of non-pregnant adult, pregnant, or postpartum dams at indicated stages.

Results are expressed as the mean ± SD. (D) Breakdown of pancreases at different gestational days (GD) or post-partum days (PP) that contain only $CD90^+$ cells. (A-C) non-pregnant (n=7), pregnant (GD11 n=5, GD14 n=4, GD17 n=4), postpartum dams (PP1 n=6, PP4 n=16, PP14 n=3, PP21 n=1).

Supplementary Figure 5. The $Aldefluor^+$ cells display different degrees of heterogeneity within each sub-population. The $Aldefluor^+$ cells isolated from the pancreas of postpartum day 1 dams were stained for CD90 and Ecadherin (left column), and the localization of each sub-population (black dots) was determined on the Aldefluor plot (middle column), or on the forward and side scatter plot (right column)

Supplementary Figure 6: Gene expression analysis of different $Aldefluor^+$ cell populations. (A) Hierarchical clustering of gene expression data among the cells analyzed in this study cluster $CD90^-/Ecad^-$, $CD90^+/Ecad^-$, and $CD90^+/Ecad^+$ populations together, indicating that these three sub-populations may share the same origin. (B) Scatter plot demonstration of genes differentially expressed in $CD90^-/Ecad^-$, $CD90^+/Ecad^-$, and $CD90^+/Ecad^+$ populations compared to $CD90^-/Ecad^+$ cells. (n=3, where each sample was pooled cells from 3-5 mice).

Supplementary Figure 7: Existence of $Aldefluor^+/CD29^+/CD90^-/Ecad^-$ cells in the embryonic dorsal mesenchyme. Representative flow cytometric analysis of ALDH1-activity, or Ecadherin and CD29 expression in $Aldefluor^+$ cells from the E10.5 dorsal pancreatic mesenchyme (dm) shows the presence of $CD29^+/CD90^-/Ecad^-$ cells in the mesenchyme surrounding the dorsal pancreatic anlagen (n=3, where each sample was pooled dorsal mesenchyme from 10-15 embryos).

Supplementary Figure 8: Expansion of Aldefluor^{+} cells in DT-treated PdxCre;R26^{DTR} mice. (A) Whole-mount X-gal staining of DT-treated PdxCre;R26$^{DTR/LacZ}$ mice show that DT-treatment results into ablation of acinar and endocrine cells, while the ductal cells survive. (B) Flow cytometric analysis of ALDH1-activity, or Ecadherin expression in Aldefluor^{+} cells isolated from the pancreas of PdxCre;R26^{DTR} on days 3 post DT-treatment, showing expansion of Aldefluor^{+} cells following DT/DTR-mediated cell ablation (n=3). (C, D) Pancreatic tissues obtained from PdxCre;R26$^{DTR/mTmG}$ mice on day 3 post DT-treatment were immunostained for the endothelial marker PECAM (C), or macrophage marker F4/80 (D). The mTomato^{+}/mGFP^{+} cells did not express PECAM or F4/80, indicating that these are not endothelial cells nor macrophages. Arrows in (C) and (D) mark mTomato^{+}/mGFP^{+}/PECAM^{-}, or mTomato^{+}/mGFP^{+}/F480^{-} cells, respectively. Bars 20 μm.

SFig.1

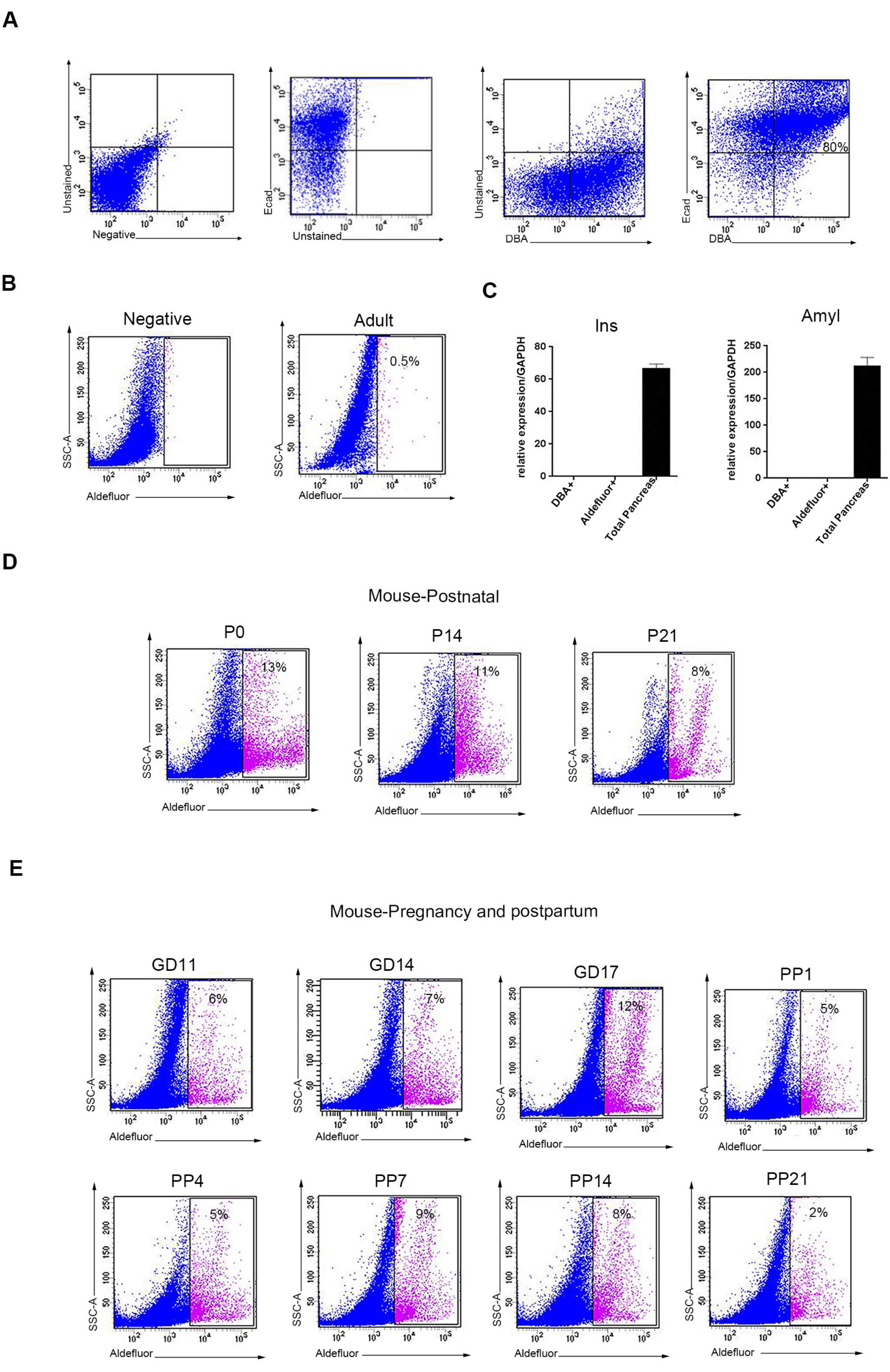
A
Negative
Unstained
Ecad
Unstained
DBA
Unstained
DBA
Ecad
80%
B
Negative
Adult
0.5%
SSC-A
Aldefluor
C
Ins
Amyl
relative expression/GAPDH
DBA+
Aldefluor+
Total Pancreas
D
Mouse-Postnatal
P0
13%
P14
11%
P21
8%
E
Mouse-Pregnancy and postpartum
GD11
6%
GD14
7%
GD17
12%
PP1
5%
PP4
5%
PP7
9%
PP14
8%
PP21
2%

SFig2

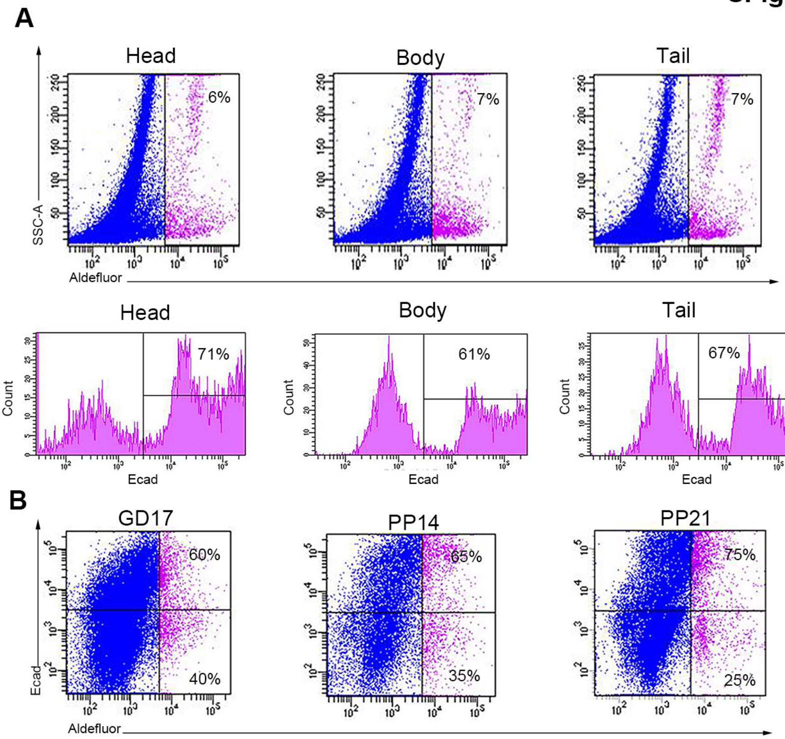

C

% Ecad-positive cells

	Control	GD11	GD14	GD17	PP1	PP4	PP14	PP21
**Median**	79	70	67	67	61	64	53.5	65
**Maximum**	93	94	80	89	83	74	78	79
**Minimum**	63	26	25	47	31	26	30	28

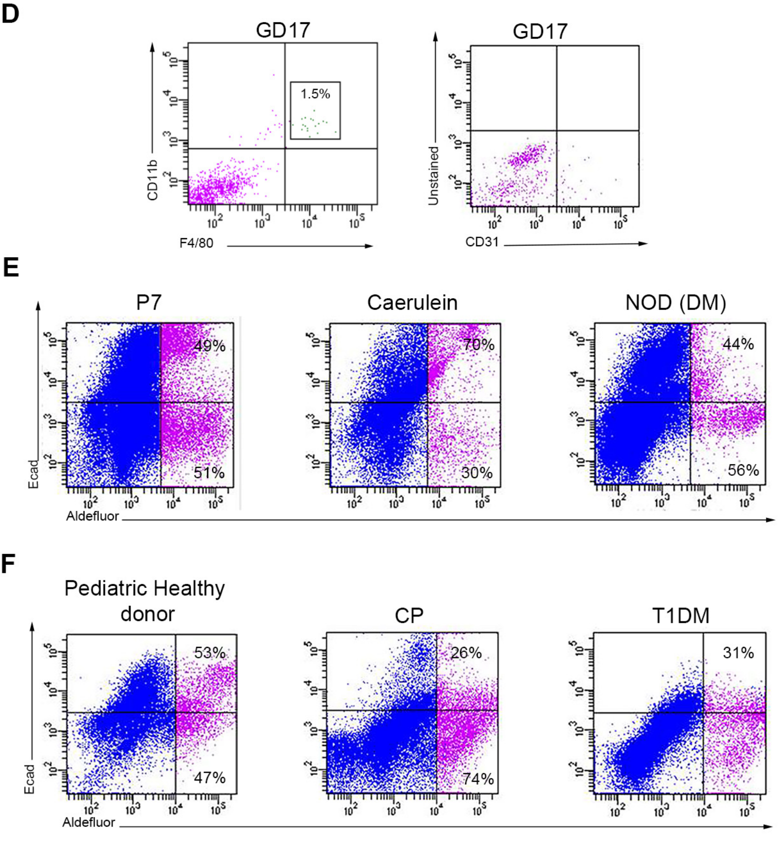

SFig 3

A

Aldefluor+ cell (Non pregnant dams)

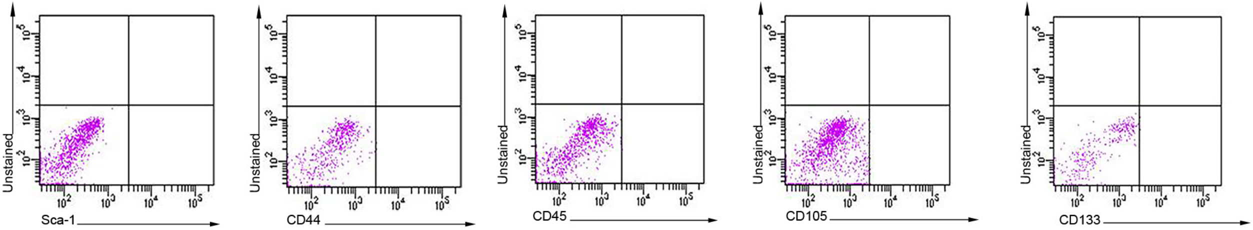

B

Aldefluor+ cell (Pregnant or postpartum dams)

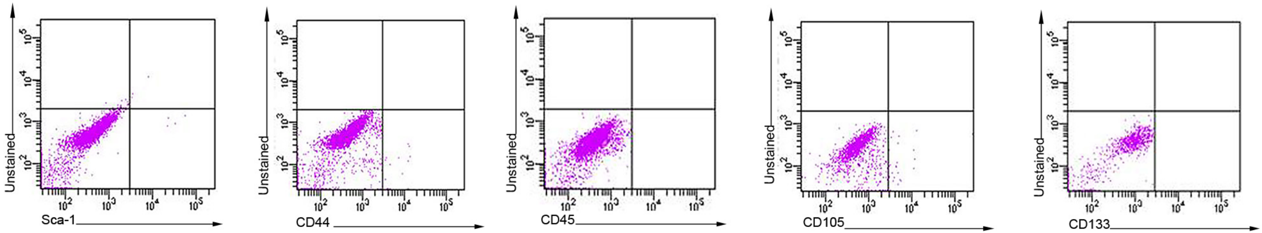

SFig4

A

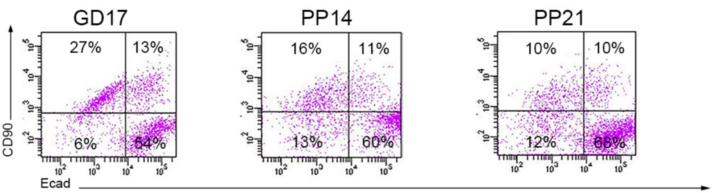

B

Number of each Cell Population (x1000)								
	**Control**	**GD11**	**GD14**	**GD17**	**PP1**	**PP4**	**PP14**	**PP21**
**CD90⁻/Ecad⁻**	21,6± 9,6	95,4± 8,4	29,0± 4,9	42,5± 9,6	45,8±27,1	80,0± 47,0	67,9± 6,7	40,4
**CD90⁺/ Ecad⁻**	----	----	99,2 ± 40,7	184,4± 86,8	58,9±12,7	64,0± 34,3	52,8±23,1	32,0
**CD90⁺/ Ecad⁺**	----	----	23,7± 12,9	101,0± 17,5	27,8±16,8	25,2± 10,7	49,9± 6,0	36,1
**CD90⁻/ Ecad⁺**	74,2± 9,6	204,7± 8,4	236,2± 48,3	294,5±103,9	112,7±27,3	180,1±55,6	311,0±23,9	225,6

C

% of each Cell Population								
	**Control**	**GD11**	**GD14**	**GD17**	**PP1**	**PP4**	**PP14**	**PP21**
**CD90⁻/Ecad⁻**	22.6 ± 10.0	31.8 ± 28.1	7.3 ± 1.3	6.8 ± 1.6	18.7 ± 11.0	22.9 ± 13.5	14.0 ± 1.4	12.1
**CD90⁺/ Ecad⁻**	----	----	25.6 ± 10.5	29.6 ± 14.0	24.0 ± 5.2	18.3 ± 9.8	11.0 ± 4.8	9.6
**CD90⁺/ Ecad⁺**	----	----	6.1 ± 3.3	16.2 ± 2.8	11.3 ± 6.8	7.2 ± 3.1	10.4 ± 1.2	10.8
**CD90⁻/ Ecad⁺**	77.4 ± 10.0	68.2 ± 28.1	61.0 ± 12.4	47.4 ± 16.7	46.0 ± 11.2	51.6 ± 15.9	64.6 ± 5.0	67.5

D

Presence of Aldefluor+/CD90+/Ecad+ cells		
**GD11**	(0/5)	0%
**GD14**	(4/7)	57%
**GD17**	(4/5)	80%
**PP1**	(6/8)	75%
**PP4**	(16/27)	59%
**PP21**	(1/3)	33%

SFig.5

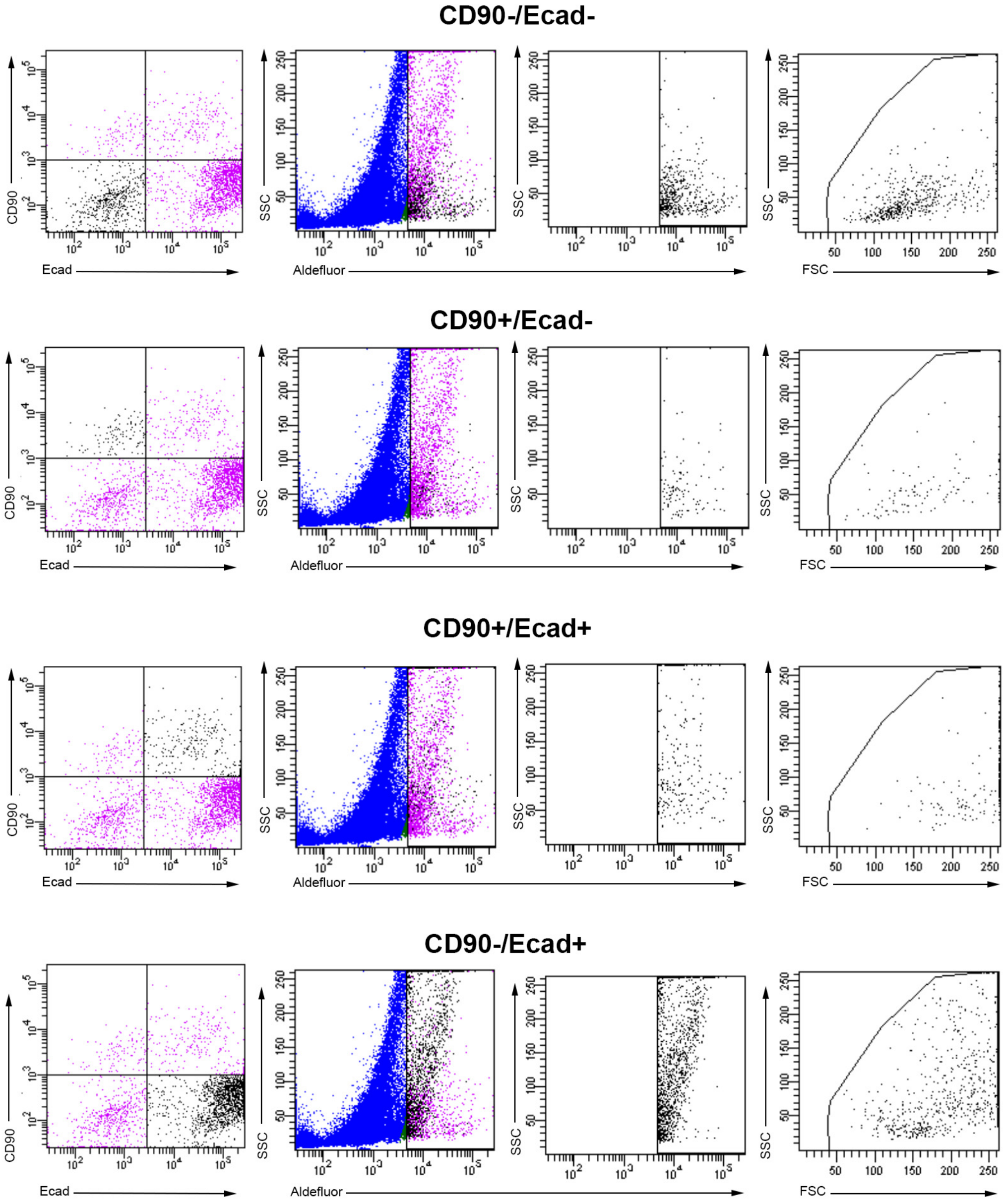
CD90-/Ecad-
CD90+/Ecad-
CD90+/Ecad+
CD90-/Ecad+
CD90
Ecad
SSC
Aldefluor
FSC

SFig6

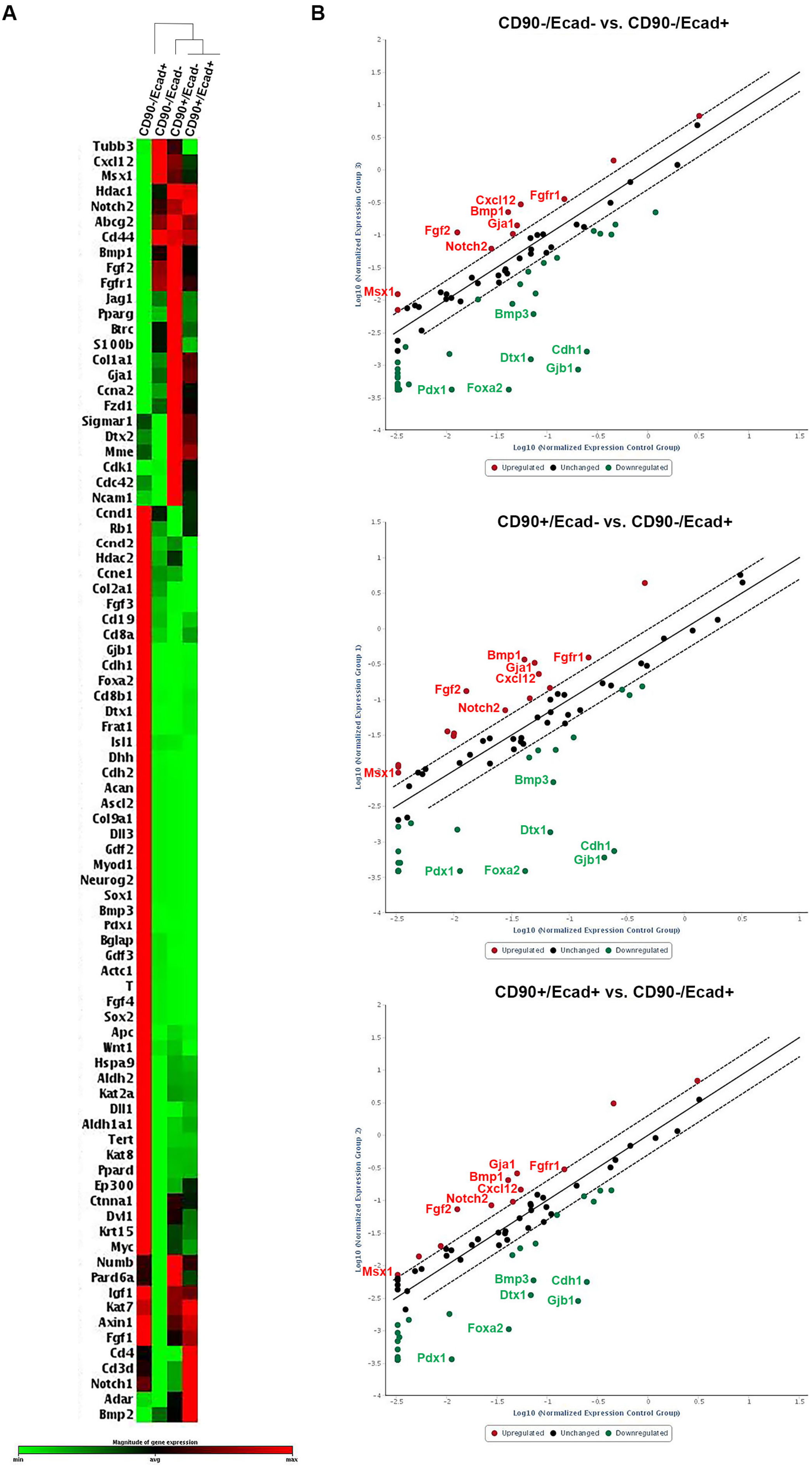
A
CD90-/Ecad+
CD90-/Ecad-
CD90+/Ecad-
CD90+/Ecad+
Tubb3
Cxcl12
Msx1
Hdac1
Notch2
Abcg2
Cd44
Bmp1
Fgf2
Fgfr1
Jag1
Pparg
Btrc
S100b
Col1a1
Gja1
Ccna2
Fzd1
Sigmar1
Dtx2
Mme
Cdk1
Cdc42
Ncam1
Ccnd1
Rb1
Ccnd2
Hdac2
Ccne1
Col2a1
Fgf3
Cd19
Cd8a
Gjb1
Cdh1
Foxa2
Cd8b1
Dtx1
Frat1
Isl1
Dhh
Cdh2
Acan
Ascl2
Col9a1
Dll3
Gdf2
Myod1
Neurog2
Sox1
Bmp3
Pdx1
Bglap
Gdf3
Actc1
T
Fgf4
Sox2
Apc
Wnt1
Hspa9
Aldh2
Kat2a
Dll1
Aldh1a1
Tert
Kat8
Ppard
Ep300
Ctnna1
Dvl1
Krt15
Myc
Numb
Pard6a
Igf1
Kat7
Axin1
Fgf1
Cd4
Cd3d
Notch1
Adar
Bmp2
Magnitude of gene expression
min
avg
max
B
CD90-/Ecad- vs. CD90-/Ecad+
Log10 (Normalized Expression Group 3)
Log10 (Normalized Expression Control Group)
Fgfr1
Cxcl12
Bmp1
Gja1
Fgf2
Notch2
Msx1
Bmp3
Cdh1
Dtx1
Gjb1
Pdx1
Foxa2
Upregulated
Unchanged
Downregulated
CD90+/Ecad- vs. CD90-/Ecad+
Log10 (Normalized Expression Group 1)
Log10 (Normalized Expression Control Group)
Bmp1
Fgfr1
Gja1
Cxcl12
Fgf2
Notch2
Msx1
Bmp3
Dtx1
Cdh1
Gjb1
Pdx1
Foxa2
Upregulated
Unchanged
Downregulated
CD90+/Ecad+ vs. CD90-/Ecad+
Log10 (Normalized Expression Group 2)
Log10 (Normalized Expression Control Group)
Gja1
Fgfr1
Bmp1
Cxcl12
Notch2
Fgf2
Msx1
Bmp3
Cdh1
Dtx1
Gjb1
Foxa2
Pdx1
Upregulated
Unchanged
Downregulated

SFig. 7

E10.5 (dm)

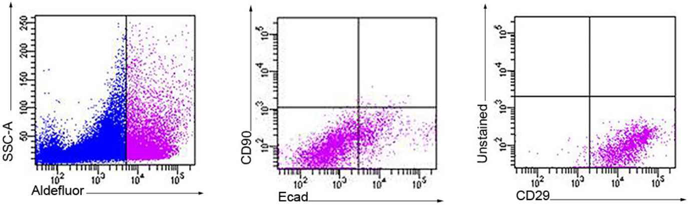

SFig 8

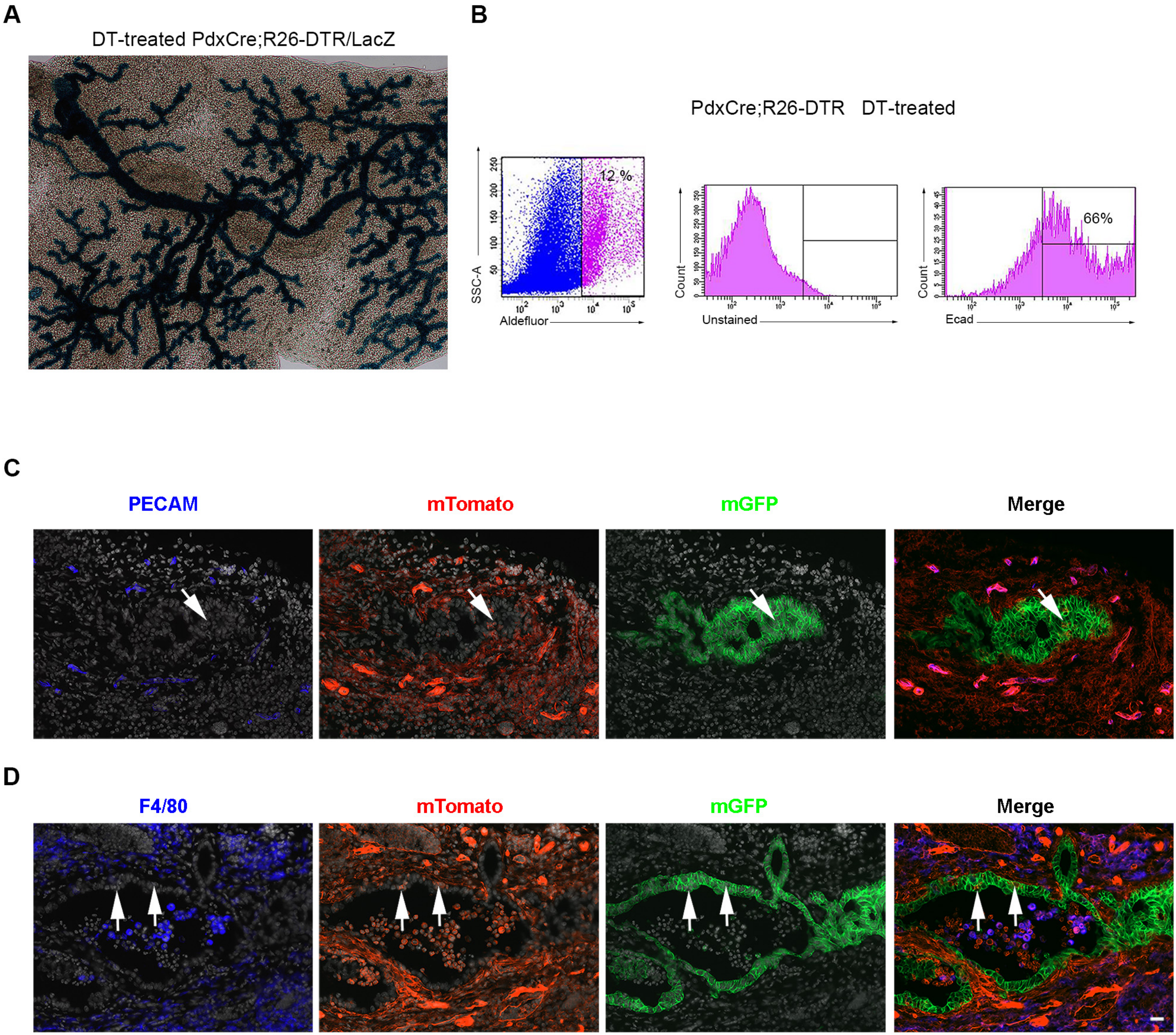

A
DT-treated PdxCre;R26-DTR/LacZ
B
PdxCre;R26-DTR DT-treated
12 %
SSC-A
Aldefluor
Count
Unstained
66%
Count
Ecad
C
PECAM
mTomato
mGFP
Merge
D
F4/80
mTomato
mGFP
Merge

Supplementary Methods

Supplementary Table 1: List of antibodies

Antigen	Species	Comapany	Cat. No.	Dilution
Amylase	goat	Santa Cruz	SC-12281	1:300
BrdU	mouse	BD Horizon	563445	5 µl/test
CD11b-APC	mouse	Miltenyi	130-091-241	1:20 (Flow)
CD11b-VioBlue	mouse	Miltenyi	130-097-336	1:20 (Flow)
CD326-biotin	mouse	Miltenyi	130-101-859	1:10 (Flow)
CD326-Vioblue	mouse	Miltenyi	130-102-421	1:10 (Flow)
CD29-APC	mouse	Miltenyi	130-102-557	1:10 (Flow)
CD31 (PECAM)	rat	BD	BD550274	1:100 (Flow)<br>1:50 (IF)
CD44-APC	mouse	Miltenyi	130-102-563	1:10 (Flow)
CD45-APC	mouse	Miltenyi	130-102-544	1:10 (Flow)
CD90.1-VioBlue	mouse	Miltenyi	130-102-637	1:10 (Flow)
CD105-APC	mouse	Miltenyi	130-102-495	1:10 (Flow)
CD133-APC	mouse	Miltenyi	130-102-197	1:10 (Flow)
Cytokeratin	rabbit	Dako	Z0622	1:100 (IF)
DBA	Fitc	Vector Lab	FL1031-2	1:100 (IF)
DBA	biotin	Vector Lab	B1035	1:100 (Flow, IF)
DCLK1	rabbit	Abcam	ab37994	1:33 (Flow)
Ecad	goat	R&D	AF748	1:200 (IF)<br>1:100 (Flow)
F4/80	rat	Invitrogen	MF48000	1:100 (IF)
F/480 eFluor 660	rat	eBioscience	50-4801-82	1:100 (Flow)
Insulin	guinea pig	DAKO	Millipore	1:1000
PDX1	goat	Abcam	Ab47383	1:10000 (IF)
PSA-NCAM-APC	mouse	Miltenyi	130-093-273	1:10 (Flow)
Sca-1-APC	mouse	Miltenyi	130-102-343	1:10 (Flow)

All of the secondary antibodies used for Immunostaining were purchased from Jackson ImmunoResearch Laboratories: biotin-conjugated anti-rabbit (1:500), biotin-conjugated anti-rat (1:500), biotin conjugated anti-guinea pig (1:500), biotin-conjugated anti-goat (1:250); Cy2-conjugated streptavidin (1:500); Cy3-conjugated streptavidin (1:500); Cy5-conjugated streptavidin (1:100); and Cy2- and Cy3- anti-guinea pig, anti-rabbit, anti-rat, anti-goat (all 1:300). For flow cytometry, secondary antibodies used were Cy5 anti-goat, anti-rat, anti-rabbit (1:100), Cy5-conjugated streptavidin (1:100) and Donkey anti-Goat IgG (H+L) Alexa Fluor® 350 conjugate (Thermo fisher).

Supplementary Table 2: List of primers

**Primers**				
Amy	mouse	Qiagen	Mm_Amy2a5_2_SG	QT01766961
GAPDH	mouse	Qiagen	Mm_Gapdh_3_SG	QT01658692
Ins2	mouse	Qiagen	Mm_Ins2_1_SG	QT00114289
